# Critical Sites on Ostreolysin Are Responsible for Interaction with Cytoskeletal Proteins

**DOI:** 10.3390/biomedicines10102442

**Published:** 2022-09-30

**Authors:** Nastacia Adler Berke, Antonella Di Pizio, Timothy D. Vaden, Irit Shoval, Ofer Gover, Daniel Waiger, Gili Solomon, Kristina Sepčić, Betty Schwartz

**Affiliations:** 1 The Robert H. Smith Faculty of Agriculture, Institute of Biochemistry, Food Science and Nutrition, Food and Environment, School of Nutritional Sciences, The Hebrew University of Jerusalem, Rehovot 7610001, Israel; 2 Leibniz Institute for Food Systems Biology at the Technical University of Munich, 85354 Freising, Germany; 3 Department of Chemistry and Biochemistry, Rowan University, Glassboro, NJ 08028, USA; 4 Light Microscopy Unit, Faculty of Life Sciences, Bar-Ilan University, Ramat Gan 5290002, Israel; 5 Department of Biology, Biotechnical Faculty, University of Ljubljana, 1000 Ljubljana, Slovenia

**Keywords:** ostreolysin, tubulin, adipocyte, differentiation

## Abstract

We explored the structural features of recombinant ostreolysin A (rOlyA), a protein produced by *Pleurotus ostreatus* and responsible for binding to α/β-tubulin. We found that rOlyA cell internalization is essential for the induction of adipocyte-associated activity, which is mediated by the interaction of rOlyA and microtubule proteins. We created different point mutations at conserved tryptophan (W) sites in rOlyA and analyzed their biological activity in HIB-1B preadipocytes. We demonstrated that the protein’s cell-internalization ability and the differentiated phenotype induced, such as small lipid-droplet formation and gene expression of mitogenesis activity, were impaired in point-mutated proteins W96A and W28A, where W was converted to alanine (A). We also showed that an rOlyA homologue, OlyA6 complexed with mCherry, cannot bind to β-tubulin and does not induce mitochondrial biosynthesis-associated markers, suggesting that the OlyA6 region masked by mCherry is involved in β-tubulin binding. Protein–protein docking simulations were carried out to investigate the binding mode of rOlyA with β-tubulin. Taken together, we identified functional sites in rOlyA that are essential for its binding to β-tubulin and its adipocyte-associated biological activity.

## 1. Introduction

Proteins belonging to the aegerolysin protein family, synthesized by oyster mushrooms (*Pleurotus* spp.), are small (~15 kD) acidic proteins with a high affinity for membrane lipids and lipid domains. Several aegerolysin genes have been shown to be highly expressed in *Pleurotus* mushrooms, and six of their protein products have been studied [1]). Among these six aegerolysins, ostreolysin A6 (OlyA6) and its homologue recombinant OlyA (rOlyA) [2], both produced by *P. ostreatus* and differing in only one conservative amino acid substitution at position 51 (valine in OlyA6 vs. isoleucine in rOlyA), have been shown to interact specifically with lipid rafts—membrane domains that are enriched in both cholesterol and sphingomyelin (SM) [3,4,5]. Lipid rafts are involved in numerous cellular functions, including signaling, trafficking, adhesion, migration and growth, all of which may involve interactions between rafts and myriad cytoskeletal proteins. A considerable amount of data has shown that microtubules and the actin cytoskeleton are intimately associated with lipid rafts [6]. Interactions of caveolae with cytoskeletal components regulate endothelial trafficking and endocytosis [6,7]. Both rOlyA and OlyA6 are internalized via the caveolin-1-dependent endocytic pathway into the cytosol of HCT116 and MDCK cells, respectively [5,8]. Caveolins act as scaffolding proteins to cluster and regulate various signaling molecules targeted to the caveolae and activate cellular events [9]. A fluorescent recombinant OlyA6 tagged at the C-terminus with mCherry (OlyA6-mCherry) was further shown to be a potential marker for visualization of cholesterol/SM-rich membrane domains, such as lipid rafts, in live mammalian cells [5]. The same ability was shown for the C-terminal-tagged variant of pleurotolysin A2 (PlyA2), a protein derived from *Pleurotus eryngii* (PlyA2-EGFP) [10].

Tryptophan-rich regions have been reported to be responsible for the initial attachment to the membrane of several pore-forming proteins [11,12,13]. Tryptophan plays a key role in promoting interfacial binding to anionic interfaces, such as bacterial membranes [14]. Point mutations of tryptophan (W) to alanine (A) in PlyA2 also demonstrated the importance of C-terminal tryptophan residues for SM/cholesterol binding [10].

We previously demonstrated that rOlyA has the unique ability to induce cell differentiation in HIB-1B preadipocytes associated with the typical formation of small lipid droplets within 24 h, a process that usually takes at least 8 days in differentiation media containing several drugs and hormones [2]. Exposure of HIB-1B brown preadipocytes to rOlyA allowed us to measure changes in expression of genes associated with brown adipocyte differentiation and related to cellular metabolic activities, and even included modifications in cytoskeletal proteins. In that previous study, it was concluded that rOlyA interacts with the cytoskeleton, particularly with β-tubulin, and that this interaction is critical for the biological activity that induces the process of adipocyte differentiation [2]. In addition, we reported analyses of the secondary structure of rOlyA using biophysical and bioinformatics tools [2], results which were recently confirmed by the resolved crystal structure of OlyA6 [15]. However, the functional sites and domains involved in rOlyA’s biological activity and the domains associated with the tubulin interaction still need to be characterized.

The present study aimed to examine the contribution of individual tryptophan residues to the structure of rOlyA, investigate their role in interactions with cytoskeleton-associated proteins and assess their contribution to the biological function of rOlyA in brown preadipocytes. As a tool, we also used OlyA6-mCherry, which is a marker for SM/cholesterol-rich membrane domains [5]. OlyA6-mCherry can bind to the membrane and be internalized into the cell, enabling us to use the fluorescent tag as a tool to understand the functional region of OlyA6. Here, for the first time, we evaluated the biological activity of OlyA6-mCherry in HIB-1B preadipocytes and tested OlyA6-mCherry’s binding ability to α/β-tubulin. Results from these experiments, in combination with rOlyA–tubulin docking simulations, pinpoint the rOlyA region responsible for its bioactivity.

## 2. Materials and Methods

### 2.1. Construction of Expression Vector

Point-mutated genes were designed according to the coding and protein sequences of rOlyA from ostreolysin derived from *P. ostreatus* cDNA that was originally obtained by PCR as previously reported [8]. Single amino acid mutations were introduced according to this sequence. The plasmid containing rOly (pTRC99) was sequenced and aligned with KC012711.1 from NCBI GenBank. For each mutant, lyophilized pUC57-Amp plasmid was produced containing a sequence with a synthetic construct, the cloning prokaryotic expression vector pTRC99a. Plasmid pTRC99a:ECD [16] was extracted, digested with *NcoI* and *XbaI* restriction enzymes and purified in an agarose gel. The digested and purified *Oly* mutant gene products (417 bp) were cloned into linearized empty pTRC99a vector. This construct was transfected into *E. coli* strain DH5α and isolated colonies expressed the mutant proteins. The resulting plasmids were confirmed by digestion with *XbaI* and *NcoI*.

### 2.2. Expression and Purification of Recombinant Mutant Proteins and OlyA6-mCherry 

Mutant protein expression and purification procedures were performed as previously described [2]. Protein concentration was determined by absorbance at 280 nm using a microvolume spectrophotometer (GeneQuant100, Biochrom, Cambridge, UK). Protein sizes and purities were determined by SDS-PAGE on homogeneous 10% acrylamide gels stained with Coomassie blue. OlyA6-mCherry was produced as described in Skočaj et al., 2014 [5].

### 2.3. Determination of CD Spectra

CD spectra were recorded using a J-810 spectropolarimeter (Jasco, Easton, MD, USA) in a 0.1-cm quartz cuvette for far-ultraviolet CD spectroscopy. Spectra were collected over 190–260 nm at 25 °C. Lyophilized rOlyA, OlyA6-mCherry, and mutant proteins W28A and W96A were dissolved in water to 20 μM concentrations. The CD spectra were measured in five repetitions to obtain an average spectrum for each protein.

### 2.4. Cell Lines and Culture Conditions

HIB-1B cells were obtained from ATCC and maintained in DMEM (Sigma, St. Louis, MO, USA, Cat#D5796) supplemented with 10% (*v*/*v*) fetal bovine serum (Biological Industries, HaEmek, Israel, Cat#04-007-1A) and 0.2% (*v*/*v*) penicillin–streptomycin–nystatin (Bio-Lab, Jerusalem, Israel, Cat#943130203800) (growth medium). Cells were cultured in 5% CO_2_ in a humidified atmosphere at 37 °C. When cells reached 80–90% confluence, they were trypsinized using trypsin solution for approximately 3 min at 37 °C. Detached cells were resuspended in growth medium and seeded in plates. HIB1B cells were routinely tested for mycoplasma contamination.

### 2.5. Treatment of HIB-1B Cells

HIB-1B cells were plated in 6-well plates (Thermo Fisher Scientific, Carlsbad, CA, USA, Cat#140675) (2.0 × 10^5^ cells) and allowed to adhere overnight. Cells were treated with growth medium alone or supplemented with rOlyA or rOlyA mutants at a concentration of 10 μg mL^−1^. On day 2, HIB-1B cells were preincubated with rOlyA or rOlyA mutants at a concentration of 10 μg mL^−1^ or higher for the dose–response experiment. Tris-NaCl pH 8.0, the elution buffer for the mutants, was tested on cells in the same volume as the mutant proteins. For inhibition of internalization of rOlyA into HIB-1B cells, we used methyl-β-cyclodextrin (MβCD, Cat# 128446-36-6), which was obtained from Sigma Aldrich Fine Chemicals, Israel. Cells were seeded in 6-well plates at a density of (2.0 × 10^5^ cells) and allowed to adhere overnight. Then, the media were aspirated and replaced with the test solution containing only media or media containing MβCD dissolved in HEPES 20 mM. The endocytic pathway inhibitor MβCD was tested at 1.25 mM for 4.5 h. Then, the cells were harvested, seeded in coverslips, treated with rOlyA for 2 h and then underwent microscopic visualization. Three independent experiments were carried out.

### 2.6. Sandwich Solid-Phase Binding Assay and Tubulin Polymerization Assay

To analyze the direct binding of the proteins of interest to α/β-tubulin and polymerized α/β-tubulin, solid-phase binding assays were performed as described previously [2]. Briefly, ELISA plates (Thermo Fisher Scientific, Cat#158348) were coated with 50 ng human β-tubulin 20 µg (0.1 mg mL^−1^), Abcam ab70187 or human α-tubulin 2 µg (0.06 µg µL^−1^ H00007846-P01) in 100 μL carbonate-bicarbonate buffer (pH 9.5) for 1 h at 37 °C. After washing, blocking was performed with 3% (*w*/*v*) bovine serum albumin (BSA) (Sigma, Cat#A9647) in 200 μL Tris-buffered saline (TBS) at 37 °C for 1 h. Wells were then washed with TBSTx1 (TBS and 0.1% Tween-20). Proteins of interest were added at 1:2 dilutions in 100 μL TBS, with rOlyA added at 10 ng well^−1^ up to 50 ng well^−1^; mutants W28A and W96A at 50 ng well^−1^, 75 ng well^−1^ and 100 ng well^−1^; and OlyA6-mCherry at 50 ng well^−1^ and 150 ng well^−1^, then incubated for 1 h at 37 °C and washed three times with TBSTx1. Except in the case of OlyA6-mCherry, each well was treated with 100 μL rabbit anti-rOlyA diluted 1:2500 in TBS and incubated. The wells were washed with TBSTx1. The primary antibodies, rabbit anti-rOlyA in TBSx1, were incubated for 1 h at 37 °C, followed by three washes in TBSTx1. The secondary antibodies, goat anti-rabbit IgG-HRP in TBSx1, were incubated for 1 h at 37 °C, followed by three washes in TBSTx1. Then, 100 μL substrate (TMB, SouthernBiotech, Birmingham, AL, USA, Cat#0410-1) was added. After formation of a color reaction, TMB stop solution (2 M H_2_SO_4_) was added. Absorbance at 450 nm was measured using an ELX 808 ultra-microplate reader (BioTek Instruments, London, UK) with KCJunior software (York, UK). Affinity was evaluated by column plot.

The tubulin polymerization assay was based on the original method published by Lee et al. (1977) [17]. The standard polymerization reaction contained 100 μL volume of 3 mg/mL α/β-tubulin in 80 mM PIPES pH 6.9, 0.5 mM EGTA, 2 mM MgCl_2_, 1 mM GTP and 10% glycerol. Polymerization was started with incubation at 37 °C and followed by absorbance readings at 340 nm. Under these conditions, polymerization reached a maximal OD340 between 0.18–0.28 nm within 30 min and, at this time, polymerized α/β-tubulin was harvested. ELISA plates were then coated with aliquots of 50 ng polymerized tubulin and the sandwich solid-phase binding assay performed as described above.

### 2.7. Microscopy—Visualization of Colocalization of Tubulin Filaments and the Proteins of Interest in HIB-1B Cells

HIB-1B preadipocytes were seeded on coverslips and allowed to adhere overnight. They were then incubated with rOlyA or mutant proteins (10 μg mL^−1^) or without treatment (control) for 90 min, 7 h or 25 h and then washed with prewarmed PBS (Bio-Lab, Cat#001623237500), fixed with 4% paraformaldehyde [Electron Microscopy Sciences (EMS), Hatfield, PA, USA, Cat#15710] in PBS for 20 min, washed three times with ice-cold PBS, permeabilized with 0.2% (*w*/*v*) Tween 20–PBS solution for 10 min on ice, then washed three times for 5 min. Cells were covered with blocking solution, 10% (*w*/*v*) goat serum (Biological Industries, Cat#04-009-1A), in PBST (phosphate-buffered saline with Tween^®^ detergent) buffer for 30 min; the blocking buffer was removed, and the diluted primary antibodies (in blocking buffer) were layered on the cover slips. First incubation was with antibodies against rOlyA (1:500) (designed and produced in our laboratory) diluted in blocking solution at 4 °C overnight. Anti-α tubulin (1:200; ab7291) and anti-β-tubulin (1:200, ab6046) were diluted in the blocking solution. Cells were incubated with primary antibody solution for 1 h at room temperature and then washed. Alexa Fluor secondary antibodies were added (1:500) in fresh blocking solution for 60 min at room temperature, followed by PBS washes. Cells were stained with DAPI, mounted in Fluoro-Gel (EMS, Cat#17985-10) and visualized by fluorescence laser scanning confocal microscopy with a Leica SP8 according to the following parameters: magnification—60X water; tubulin—Alexa Fluor 488, excitation 490 nm, emission 525 nm; caveolin-1—Alexa Fluor 488, excitation 490 nm, emission 525 nm; rOlyA—Alexa Fluor 594, excitation 590 nm, emission 617 nm; OlyA6-mCherry—excitation 587 nm, emission 610 nm; actin iFluor (phalloidin) 647—excitation 650 nm, emission 665 nm; DAPI—excitation 350 nm, emission 470 nm.

### 2.8. Image Analysis

All imaging experiments were repeated at least three times, and a minimum of seven cells were analyzed per replicate. Three-dimensional (3D) confocal stacks of immunofluorescence were background-subtracted. Representative cells were selected from a random field of view on the coverslip. In addition, we automated our image analysis pipeline to ensure uniformity among all imaging processes. The resulting images were analyzed with Imaris software (version 8.4.1; Bitplane AG, Oxford Instruments, UK). Imaris was used to generate spots for the OlyA-containing proteins and tubulin filaments. Analysis flow was as follows: as tubulin is filamentous, we used the Imaris Filament module to create a filament object for the α- or β-tubulin stain (autopath algorithm, no loops). Next, we created a surface object for the nuclei using DAPI staining to get the total cell count in the image. Finally, we used the spot tool to identify OlyA6-mCherry. Once all objects were identified, we used the Find Spots Close to Filaments extension to identify the spots that colocolize with the tubulin filaments. All objects were segmented using the same parameters. The colocalized spots were defined as having a distance ≤0.2 µm, which included all protein spots that were closely attached to the tubulin filaments. Finally, the ratio between close protein spots and distant protein spots was calculated to produce the rate of rOlyA and tubulin colocalization.

### 2.9. Single-Molecule Localization Microscopy 

dSTORM data were acquired on a Nanoimager S from Oxford Nanoimaging (ONI, Oxford, UK) equipped with an Olympus 100X, 1.4 NA oil-immersion objective, an XYZ closed-loop piezo stage and laser lines: 488 nm and 640 nm (Edmund Optics Inc., Barrington, NJ, USA). Fluorescence emission was detected using an sCMOS camera (ORCA Flash 4, Hamamatsu, Shizuoka, Japan) with 488–551 nm and 576–620 nm bandpass filters in channel 1 and a 666–705 nm bandpass filter in channel 2. Samples were imaged in ONI’s BCubed imaging buffer. Data were processed with NimOS software from ONI.

### 2.10. RNA Extraction and Reverse Transcription

Cells were treated as described in the Cell lines and culture conditions section. After 24 and 48 h incubation with rOlyA or mutants, 0.6 mL TRI-reagent (Sigma) was added to each sample and cells were collected into Eppendorf tubes. Then, 0.2 mL chloroform was added per 1 mL of TRI-reagent and vortexed vigorously. Samples were incubated for 2–3 min on ice, then centrifuged for 15 min at 12,500× *g* at 4 °C (Eppendorf 5430R centrifuge, Hamburg, Germany). The aqueous phase containing the RNA, ca. 250 μL, was collected in a new tube. RNA was further purified using the PureLink RNA mini kit according to the manufacturer’s instructions (Thermo Fisher Scientific, Cat#12183018A). RNA concentration was determined using a NanoDrop 2000 spectrophotometer (NanoDrop Technologies, Montchanin, DE, USA). Total RNA was treated with DNase I using TURBO DNase (Thermo Fisher Scientific, Cat#AM1907) for 30 min at 37 °C, followed by RNase inhibitor (Thermo Fisher Scientific, Cat#EO0381). RNA (1 μg) was used for cDNA synthesis with the qScript cDNA Synthesis kit, according to the manufacturer’s protocol (Quantabio, Beverly, MA, USA).

### 2.11. Real-Time PCR

Real-time PCR relative quantification analysis using the ΔΔCT method was performed on a QuantStudio 1 instrument (Applied Biosystems, Waltham, MA, USA) with the Fast SYBR Green Master Mix (Applied Biosystems). Each sample was analyzed in triplicate with cDNA corresponding to 25 ng template RNA and a final 0.4 μm of each primer set. Cycling conditions were set at 95 °C for 10 min, followed by 40 cycles at 95 °C for 15 s and 60 °C for 1 min. All real-time PCR data were normalized to the housekeeping gene *RPL 41* [18] using a standard curve.

### 2.12. Protein Purification from Cells

Cells were treated as described in the Cell lines and culture conditions section. After incubation with rOlyA or its mutants (10 μg mL^−1^) for 7 or 25 h, plates were washed twice in ice-cold PBS and 100 μL lysis buffer added to each well. After 10 min incubation, cells were collected by scraping into cold Eppendorf tubes. After lysing, insoluble material was spun down at 4 °C at maximum speed for 10 min (Eppendorf 5430R centrifuge, Merck & Co. Herzliya Pituach, Israel). Supernatant containing the soluble protein lysates from the cells was collected and used for protein quantification.

### 2.13. Protein Quantification

Protein concentration was determined by microbicinchoninic acid-based protein assay (Thermo Fisher Scientific, Cat#23225) using BSA as the standard. Protein concentrations were assessed at 550 nm in the ELX 808 ultra-microplate reader using KCJunior software (BioTek Instruments, Santa Clara, CA, USA).

### 2.14. Western Blot Analysis

HIB-1B lysates were electrophoresed on 10–15% SDS-polyacrylamide gels and transferred to nitrocellulose membranes (Whatman, Schleicher & Schuell, Dassel, Germany). The membranes were blocked for 1 h at room temperature in TBST containing 5% (*w*/*v*) skim milk powder and incubated with primary antibodies (at different dilutions) overnight at 4 °C. Membranes were then incubated with secondary anti-rabbit antibodies for 1 h at room temperature. Proteins were visualized using Western blotting luminol reagent with a ChemiDoc^TM^ MP Imaging System (Bio-Rad Laboratories, Berkeley, CA, USA). To assess the quality and content of the loaded samples, the target proteins were normalized to β-actin.

### 2.15. Statistical Analyses

Results are expressed as mean ± s.e.m. Comparison between groups was performed by ANOVA and all-pairs Tukey-HSD. All statistical analyses were conducted with JMP (SAS Institute Inc, Cary, NC, USA), and differences were considered significant at *p* < 0.05.

### 2.16. OlyA6-mCherry Structure Modeling

mCherry homology modeling was modeled from 2H5Q.pdb (mCherry structure, res 9-228) and 3NED.pdb (mRouge structure, res 6-237). mRouge has more residues and was used as a model for regions that were not solved in the mCherry structure.

To build the model of OlyA6-mCherry, we used protein–protein docking simulations (Piper as implemented in ClusPro 2.0) [19] to sample the possible interacting modes of OlyA6 and mCherry, and loop modeling and minimization tools (Prime and Macromodel, Schrödinger) to connect the two structures. The OlyA6-mCherry complex has a linker of 12 amino acids (GSEGKGSSSGSG); we modeled OlyA6-GSE (from PDB ID: 6MYK) and GSG-mCherry (PDB ID: 2H5Q, missing residues in this structure were modeled using the mRouge structure 3NED.pdb as template) as input structures for the docking. The OlyA6 structure ends with a non-structured loop and, therefore, several conformations of this loop were used as input structures for the docking analysis. We generated 577 docking poses and, by visual inspection, we selected 71 poses that place the C-terminus of OlyA6 in close proximity to the N-terminus of mCherry to allow the link and protein binding. These 71 poses were then clustered by structural similarity into 12 poses. The representative structures of the clusters were analyzed. The final model was selected based on the involvement of W28 and W96 in the interaction and the population of the cluster. Using Prime (Schrödinger), we modeled the linker that connects the two proteins in the complex and refined the 3D model.

### 2.17. β-Tubulin–Olya6 Docking

Docking calculations were performed with β-tubulin (PDB ID: 1TUB) as the target and OlyA6 (PDB ID: 6MYI) as the ligand using ClusPro 2.0 [20], Boston University, Boston, USA. We set 30 poses as output because it has been shown that the 30 largest clusters contain at least one near-native structure [20]. These 30 poses were then minimized to a derivative convergence of 0.05 kJ mol^−1^ Å^−1^ using the Polak–Ribiere–Polyak (PRP) conjugate gradient minimization algorithm, the OPLS2005 force field and the GB/SA water-solvation model implemented in MacroModel (Schrödinger 2018). The best pose among the 30 generated docking poses was selected considering the ranking order (cluster population) and taking into consideration the fact that if OlyA6 is supposed to bind to microtubules, it cannot bind to regions of β-tubulin that are involved in heterodimerization with α-tubulin.

## 3. Results

### 3.1. Preparation of rOlyA Point Mutations W28A, W92A, W96A, W112A

**Figure 1:**
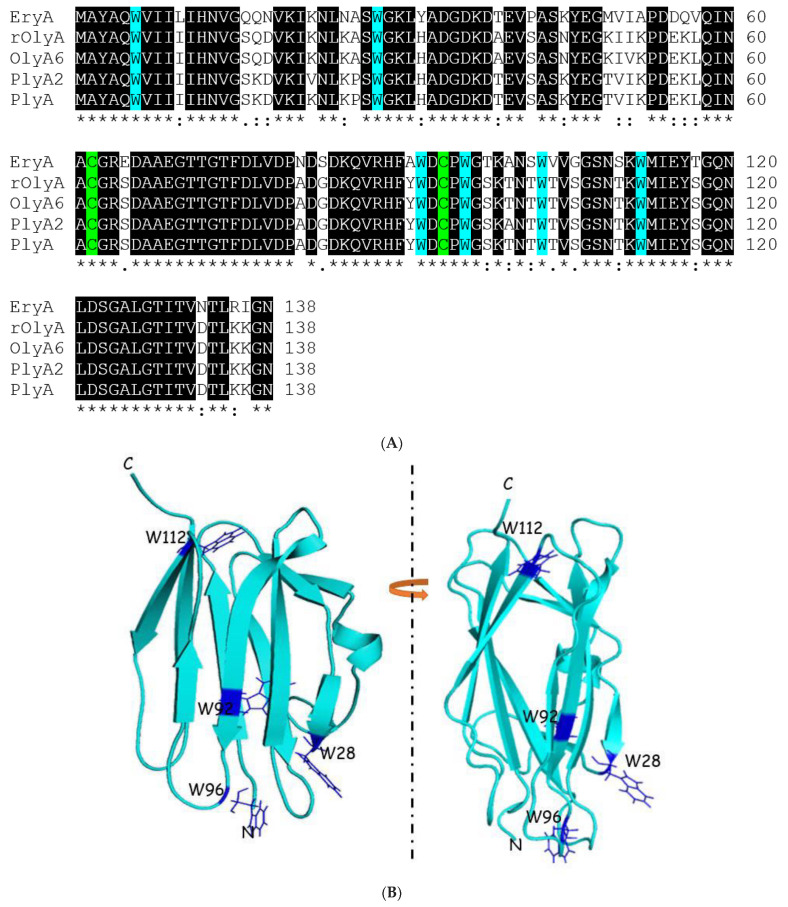

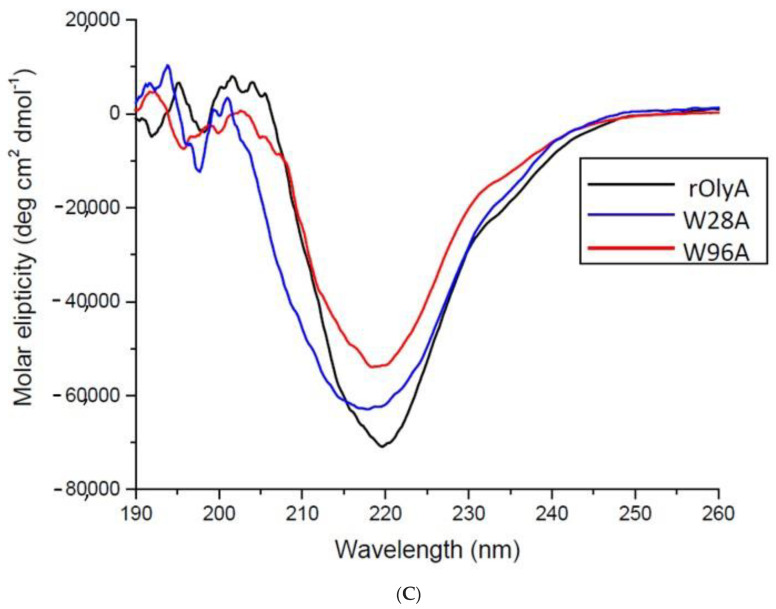
Conservation of aegerolysins and secondary structure analysis of rOlyA protein. (**A**) Alignment of the amino acid sequence (with CLUSTAL omega) of rOlyA and its homologous/orthologous proteins from the aegerolysin family. The family consists of recombinant ostreolysin A (rOlyA; GenBank entry KDQ25828) and five other members: OlyA6 (P83467), PlyA (Q8X1M9), OlyA (AAX21097), PlyA2 (BAN83906.1) and EryA (BAI45247.1). Strictly conserved residues are shown in black. Two cysteine residues were conserved in all five proteins (green). Six tryptophan residues were conserved (turquoise). Numbering is according to the rOlyA sequence. rOlyA and OlyA6 differed in only one amino acid substitution: rOlyA—isoleucine, OlyA6—valine (red box). Abbreviations are as follows: EryA—erylysin A, OlyA—ostreolysinA (*Pleuratus ostreatus*), with high similarity to PlyA, protein from *P. ostreatus* and its ortholog, PlyA2, from *P. eryngii*; rOlyA—recombinant OlyA in plasmid pTRC99A. (**B**) Cartoon representation of rOlyA model. Tryptophans for mutation are represented in blue. Positions of displayed residues as indicated. N—N-terminus; C—C-terminus. (**C**) CD spectra to estimate secondary protein structure of rOlyA, W28A and W96A demonstrate that the secondary structure of rOlyA and its mutants fits a β-sheet spectrum, since the CD curve analyses are consistent.

Our strategy to identify tryptophan residues that are important for rOlyA membrane interactions was based on previous studies conducted with its orthologue PlyA2 [10], which was shown to selectively bind membranes enriched in both cholesterol and SM, mostly found in lipid rafts. Tryptophan-rich regions have been reported in that protein and shown to be responsible for initial attachment to the membrane [13], and selected W-to-A point mutations demonstrated the importance of C-terminal tryptophan residues for membrane–protein interactions and SM/cholesterol binding [10]. rOlyA contains six tryptophan residues at positions 6, 28, 92, 96, 103 and 112 (Figure 1A). Point mutations W28A, W92A, W96A and W112A were introduced in rOlyA by replacing individual tryptophan with alanine residues. The four different point-mutated genes were cloned into expression vectors and transformed into *Escherichia coli* DH5α cells. Two mutants, W28A and W96A, were successfully expressed. Tryptophan residues 28 and 96 lie on two separate loops and are oriented outward on the N-terminus. These rOlyA mutants were soluble and were found in the supernatant fraction. The proteins were detected by Western blot using a rOlyA-specific antibodies [2,8] (see Supplementary Materials Figure S1). The two other mutants, W92A and W112A, were only identified at low concentrations in the pellets. W112A was accumulated in the inclusion bodies. W92A was expressed at very low concentrations in a constitutive fashion (not dependent on isopropyl-1-thio-β-D-galactopyranoside (IPTG) induction) but dependent on temperature and time of incubation. Residues W92 and W112 are oriented toward the beta-sandwich core of the protein (Figure 1B); hence, we assumed that their presence might be important for the stability of rOlyA and for protein folding. As a consequence, mutating those amino acids might impact the expression in host bacteria.

The supernatant fraction, containing soluble proteins W28A and W96A, was used for further purification with anion-exchange chromatography. The protein concentration was determined by absorbance at 280 nm, and monomer content was determined by gel-filtration chromatography on a size-exclusion chromatography (SEC) Superdex 75 column equilibrated with Tris-NaCl pH 8.0. Both mutants, W28A and W96A, eluted at 50 mM NaCl. The yield of the mutants was relatively low compared to that of the wild-type rOlyA. The purified recombinant protein was greater than 95% homogeneous, as determined by SDS–PAGE (15%), which showed a size identical to that of the native protein (~15 kD) obtained from a culture filtrate. A different degree of purity was achieved with the mutant derivatives compared to the wild-type protein.

To estimate whether the secondary structure of rOlyA was affected by the introduction of the point mutations (W28A, W96A), we analyzed the circular dichroism (CD) spectra of both point mutations and compared them to rOlyA CD spectra (Figure 1C). The CD spectra showed no significant changes in secondary structure for either point-mutated proteins, and the β-sheet structure appeared to be completely conserved (Figure 1C).

### 3.2. Contribution of Tryptophan Residues to the Biological Activity of rOlyA in HIB-1B Preadipocytes 

**Figure 2:**
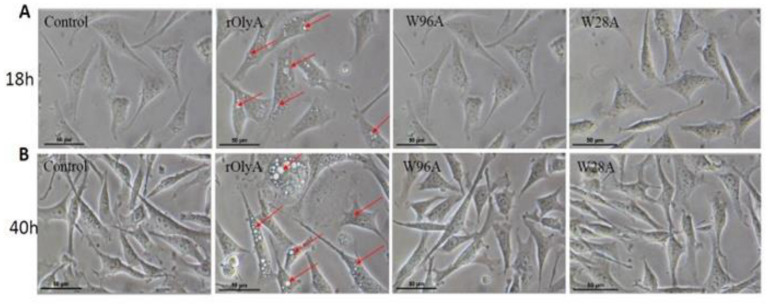
Effect of rOlyA on HIB-1B preadipocytes. Light microscopic imaging (40 after incubation with rOlyA and its mutants for 18 h (**A**) and 40 h (**B**) post-treatment. HIB-1B preadipocytes were incubated with or without 10 µg mL^−1^ rOlyA, W28A and W96A for 18 h and 40 h; microscopic images were captured. The red arrows indicate the occurrence of small lipid droplets in the rOlyA-treated group.

Our previous study in HIB-1B preadipocytes [2] (demonstrated that rOlyA (at 10 µg mL^−1^) induces striking morphological alterations consisting of the accumulation of structures resembling lipid droplets. However, the W28A and W96A mutants did not induce this phenotype after either 18 h or 40 h of incubation (Figure 2A,B, respectively). We then tested the effect of increasing concentrations of W28A and W96A (10, 30 and 50 μg mL^−1^) for 18 h (Supplementary Materials Figure S2A–H) and 40 h (Supplementary Materials Figure S2I–P). As the dose increased, a few cells showed the phenotype induced by rOlyA but at a very low frequency (Supplementary Materials Figure S2E,G,H,O,P) compared to cells treated with the wild-type rOlyA (Supplementary Materials Figure S2B,J).

### 3.3. Contribution of the Individual Tryptophan Residues to Membrane Interactions and internalization of rOlyA into HIB-1B Preadipocytes

To test the mutants’ ability to be internalized into HIB-1B cells, the cells were incubated with rOlyA or its mutants at the previously tested effective dose (10 μg mL^−1^) for 7 and 25 h. Immunodetection of cytosolic protein extracts with anti-rOlyA antibodies enabled determining whether the mutant proteins, as compared to the wild-type rOlyA, had succeeded in entering the cell’s cytosol (Supplementary Materials Figure S3). Immunoblotting of cytosolic lysates of HIB-1B cells pretreated with rOlyA detected substantial amounts of the cellular rOlyA. These data indicated that rOlyA was not degraded after 7 h of incubation with HIB-1B cells, but after 25 h of treatment, some degradation of the cellular protein occurred, as evidenced by smaller molecular weight protein species in the Western blot (Supplementary Materials Figure S3). Compared to rOlyA, cell internalization of the W96A mutant was completely abolished, and the cell-internalization ability of W28A was delayed (Supplementary Materials Figure S3). After 7 h of incubation, only rOlyA was detected in the cytosolic fractions. After 25 h, the amount of intracellular W96A was undetectable and did not change over time, but the amount of W28A in the cell increased.

### 3.4. Functionality of OlyA6-mCherry 

**Figure 3:**
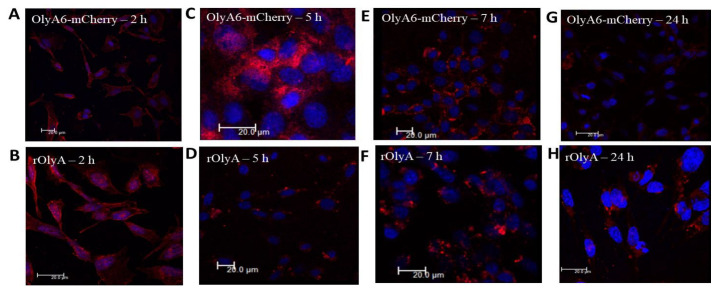
Intracellular distribution of rOlyA and OlyA6-mCherry at different time points: 2 h (**A**,**B**), 5 h (**C**,**D**), 7 h (**E**,**F**) and 24 h (**G**,**H**) in fixed HIB-1B preadipocytes. Representative images of immunofluorescence using antibodies against rOlyA (10 µg mL^−1^) immunostained with Alexa Fluor 594 or OlyA6-mCherry (30 µg mL^−1^) mCherry (610 nm). Fixed HIB-1B cells were incubated with rOlyA or OlyA6-mCherry, followed by treatment with anti-rOlyA antibodies and the secondary antibodies conjugated with Alexa Fluor 594. Scale bar = 20 µm.

To test the putative internalization activity of rOlyA for live HIB-1B preadipocytes and to assess the colocalization of rOlyA with intracellular or membrane-associated cellular organelle markers, fluorescently tagged OlyA6-mCherry was used as a complementary tool. This tagged protein has been extensively investigated as a fluorescent membrane marker since 2014 [21,22,23]. We analyzed the cell-internalization dynamics of OlyA6-mCherry with the preadipocytes and its cellular functionality compared to the untagged rOlyA detected by anti-rOlyA antibodies. We monitored the protein’s dynamics using immunofluorescence in HIB-1B cells and we tracked its progression and distribution inside the cell at different time points (2 h, 5 h, 7 h and 24 h). OlyA6-mCherry internalization and cellular distribution were similar to those of rOlyA (Figure 3A–E). Over time, these proteins accumulated in clusters close to the cell nucleus and were less distributed in the cell cytoplasm (Figure 3E,F). Interestingly, mCherry fluorescence was reduced after 24 h (Figure 3G) and showed instability after 24 h of treatment. Anti-rOlyA followed by Alexa Fluor 594 antibodies displayed improved signals compared to mCherry and, therefore, we used immunofluorescent staining with anti-rOlyA antibodies for optimal immunofluorescence detection of the tagged protein.

As seen in Supplementary Materials Figure S5, clusters of rOlyA were observed on the cell surface and within the cell when the cells were treated for 2 h with rOlyA, but when the cells were previously exposed to the inhibitor of cell internalization methyl-β-cyclodextrin (MβCD) and then treated for 2 h with rOlyA, the presence of intra-cellular rOlyA completely disappeared and only some of these clusters were detected in the cell surface, in contrast to the mutants. Fewer clusters were observed as compared to the control due to the effect of MβCD on the suitable organization of lipid rafts.

**Figure 4:**
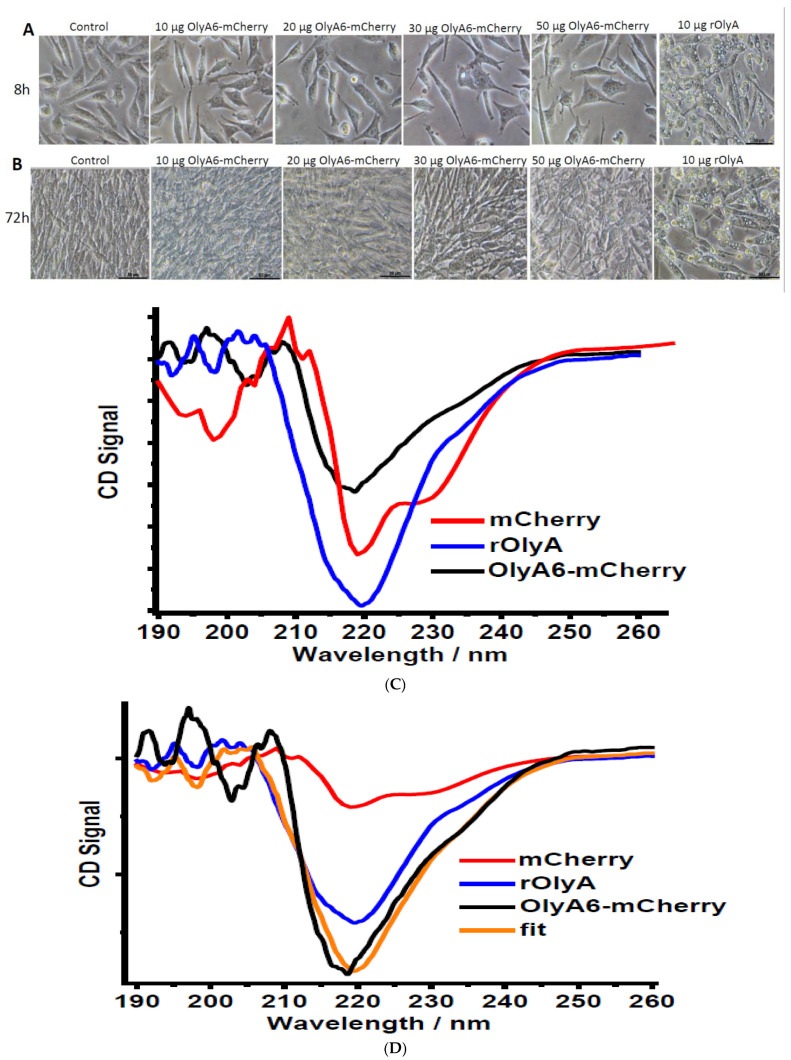

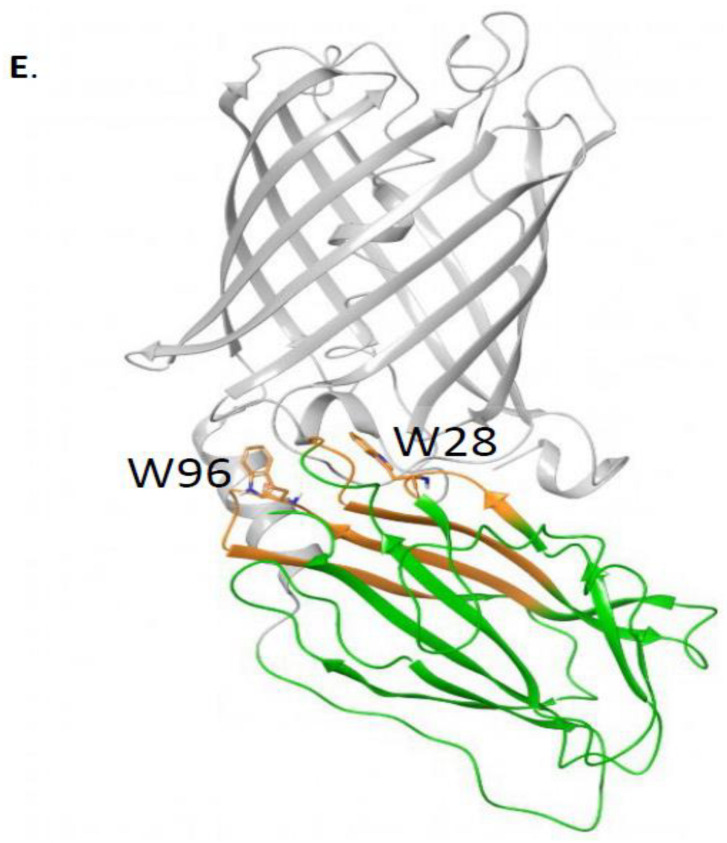
Functionality of OlyA6-mCherry. Dose response—live HIB-1B cells with rOlyA, OlyA6-mCherry incubation and imaging at two time points: 8 h (**A**) and 72 h (**B**) post-treatment. HIB-1B cells treated with OlyA6-mCherry 0 μg mL^−1^ (control), 10 μg mL^−1^, 30 μg mL^−1^ and 50 μg mL^−1^ for 8 or 72 h. Bright field (40×), scale bar = 50 µm. (**C**) CD spectra of OlyA6-mCherry, mCherry and rOlyA in the secondary-structure spectral region. The mCherry and rOlyA spectra are similar and the OlyA6-mCherry spectrum is the combination of both spectra. (**D**) OlyA6-mCherry spectra were separated into OlyA6 (red) and mCherry (black). The combined form of the spectra of OlyA6 protein and mCherry (blue) is plotted against the OlyA6-mCherry spectra (orange). Distinct 220 nm peaks of OlyA6 and mCherry indicate mainly β-sheet structures. (**E**) Structural model of OlyA6-mCherry. mCherry is represented as a gray cartoon and OlyA6 as a green cartoon. The region of OlyA6 interacting with mCherry is shown as an orange cartoon, and W28 and W96 are represented as orange sticks.

To test the functionality of OlyA6-mCherry, we treated the cells with 10, 20, 30 and 50 μg mL^−1^ of the protein at two time points: 8 h (Figure 4A) and 72 h (Figure 4B). We measured the ability of OlyA6-mCherry to induce small lipid droplets, characteristic of differentiation in HIB-1B adipocytes. The cells did not show the characteristic phenotype of differentiated cells (as opposed to when incubated with rOlyA) and grew regularly, regardless of protein concentrations. Lipid droplets were absent and cell proliferation was not altered (Figure 4A,B). These data led us to hypothesize that the mCherry tag interferes with the amino acid sequence involved in induction of the preadipocyte differentiation process by rOlyA.

### 3.5. CD Spectral Analysis of mCherry-Tagged rOlyA

To determine whether the mCherry tag influences the secondary structure of OlyA6, we used CD spectroscopy to compare the secondary structures of rOlyA and OlyA6-mCherry. The CD spectra in Figure 4C,D show the secondary structure region. mCherry exhibited a spectrum characteristic of β-barrel proteins and rOlyA showed a typical β-sheet structure. The OlyA6-mCherry spectrum was very similar to that of rOlyA, indicating a β-sheet structure. Figure 4D shows the spectra corrected for concentration. A fitted spectrum was generated by adding the rOlyA spectrum, scaled by 95%, and the mCherry spectrum, scaled by 5%. The fitted spectrum matched the experimental OlyA6-mCherry spectrum, indicating that (1) the CD spectrum of the complex is dominated by the signal from the rOlyA protein; and (2) the structure of rOlyA is clearly unchanged by complexation to mCherry. We investigated the interaction between OlyA6 and mCherry in the complex using computational tools. We performed protein–protein docking of OlyA6 and mCherry, filtered the poses that allow the interaction between the OlyA6 C-terminus and mCherry N-terminus and modeled the complexes accordingly (all details are reported in the Materials and Methods section). Different conformations were sampled and clustered. The complex model predicted that OlyA6 W28 points toward the mCherry tag, whereas W96 points out (Figure 4E). The predicted interaction surface of OlyA6 was comprised of at least residues 26–29 and 67–76. This region is masked by mCherry and, therefore, it is suggested as the region containing the residues involved in β-tubulin binding.

### 3.6. Cellular Activity: Changes in Gene-Expression Profile as a Result of rOlyA, Oly-mCherry and rOlyA Mutant Treatments 

**Figure 5:**
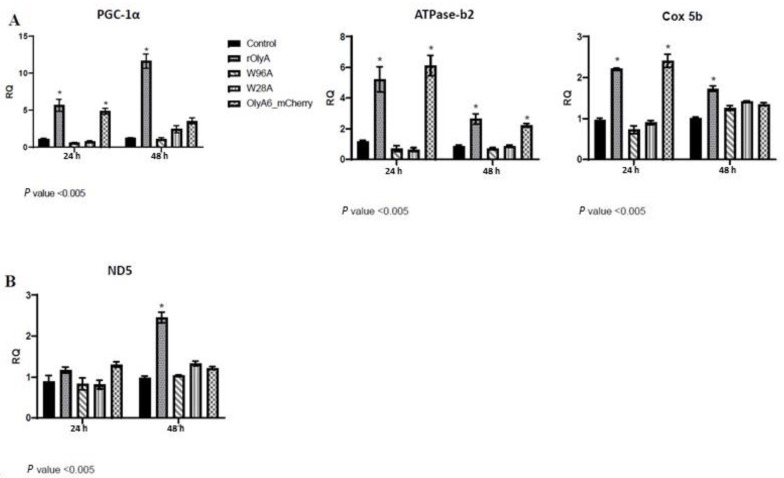
Screening for expression of mitochondrial markers using real-time RT-PCR. Preadipocyte mRNA was measured by quantitative RT-PCR as described in the Materials and Methods section. Gene expression in HIB-1B cells 24 h and 48 h after addition of proteins to growth medium: rOlyA, W96A, W28A (10 μg mL^−1^); OlyA6-mCherry (30 µg mL^−1^); and control (mock). Graph representation: y-axis—RQ (relative quantification), bars represent treatments with proteins. Each graph represents HIB-1B cell treatments for 24 h and 48 h. (**A**) Nuclear-encoded mitochondrial marker and biogenesis regulator genes, associated with energy generation and expenditure from lipid and glucose source. (**B**) Mitochondrion-encoded marker gene *ND5*. All results were normalized to *RPL 41* gene expression. All data are expressed as mean ± s.e.m., n = 4–6; * *p* < 0.0001.

To test whether the rOlyA mutants, similarly to rOlyA, can stimulate differentiation-oriented gene expression, we treated HIB-1B cells with rOlyA, OlyA6-mCherry or rOlyA mutants for 24 h and 48 h and analyzed several genes involved in metabolic activity. The nuclear receptor coactivator PPARγ coactivator-1α (*PGC-1α*) has been shown to be involved in regulation of the brown adipose tissue phenotype [2]. Figure 5A shows that rOlyA upregulated *PGC-1α* expression, whereas the W96A and W28A mutants did not. Although in rOlyA- and OlyA6-mCherry-treated cells, gene-expression levels were elevated at 24 h post-treatment, after 48 h, these levels remained significantly higher only in rOlyA-treated cells. Since *PGC-1α* is involved in mitochondrial biogenesis, we next investigated whether rOlyA, OlyA6-mCherry and rOlyA mutants affect the expression of mitochondrial genes associated with mitochondrial function in brown adipocytes and controlled by *PGC-1α*: *Cox5b* (cytochrome-c oxidase activity, hydrogen ion transmembrane transport, in mitochondrial envelope) and *ATPase-b2* (V-type proton ATPase subunit B). Figure 5A shows that rOlyA and OlyA6-mCherry significantly upregulated *ATPase-b2* and *Cox5b* genes after 24 h of treatment, whereas, after 48 h, this effect was reduced. As with *PGC-1α*, the W96A and W28A mutants did not affect these genes’ expression. Mitochondrial DNA-encoded mitochondrial biogenesis marker *ND5* gene-expression levels were only increased in rOlyA-treated cells (Figure 5B).

### 3.7. Interactions of rOlyA, OlyA6-mCherry and rOlyA Mutants with β-Tubulin 

**Figure 6:**
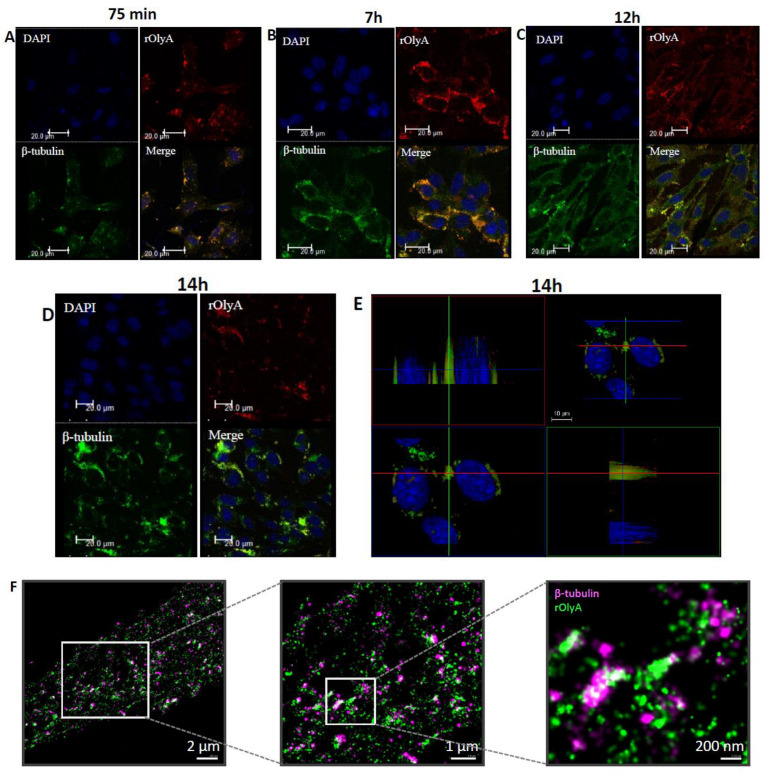

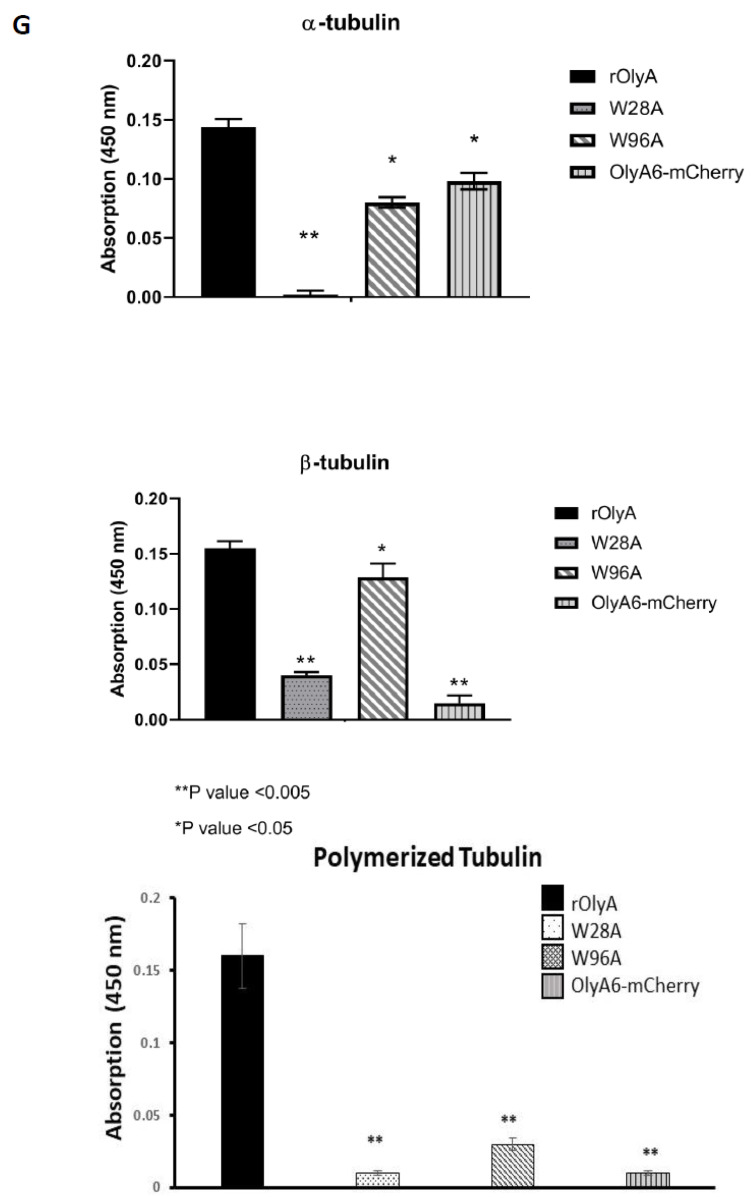
Dynamics of rOlyA protein interaction with β-tubulin after internalization into HIB-1B preadipocytes. DAPI immunostaining for colocalization of rOlyA with β-tubulin in HIB-1B cells after (**A**) 75 min, (**B**) 7 h, (**C**) 12 h, (**D**) 14 h. Zoom-in of the image to emphasize the colocalization. (**E**) Leica-3D tool (Leica Geosystems, Balgach, Switzerland) for colocalization of rOlyA with β-tubulin in HIB-1B cells at 14 h. (**F**) Super-resolution STORM imaging of rOlyA and β-tubulin protein interaction at 14 h. Images of HIB-1B preadipocytes stained with anti-β-tubulin primary antibodies and Alexa Fluor 680 (purple) or rOlyA Alexa Fluor 488 (green). Zoomed-in region of protein colocalization, tagged nanoclusters. Scale bars in zoomed-out images = 2 µm and in zoomed-in images, 1 µm and 200 nm. (**G**) Binding of rOlyA, its mutants and OlyA6-mCherry to α-tubulin (top panel) and β-tubulin (bottom panel) in a solid-phase ELISA. rOlyA and its mutants were added to immobilized α/β-tubulin and polymerized α/β-tubulin at 50 ng 100 μL^−1^ well^−1^, rOlyA 50 ng 100 μL^−1^ well^−1^ and OlyA6-mCherry 150 ng 100 μL^−1^ well^−1^, reacted with rabbit anti-rOlyA and goat anti-rabbit IgG-HRP. Protein was quantified by measuring OD at 450 nm. * *p* < 0.05, ** *p <* 0.005.

Selected *Pleurotus* aegerolysins have been shown to interact with membrane lipid rafts and to be internalized into the cell via caveolin-1-mediated endocytosis. Indeed, in MDCK cells, the highest degree of colocalization was found between OlyA6-mCherry and caveolin-1 [5]. In HCT116 cells treated for 8 h with rOlyA, caveolin-1 was also suggested to regulate its cell internalization; however, the two proteins did not colocalize [8]. We previously showed that rOlyA interacts with β-tubulin when it is internalized in HIB-1B cells [2], so the involvement of caveolin-1 in rOlyA trafficking from the membrane toward the nucleus was also assumed in the present study with the same cell type. The colocalization between the two proteins was indeed confirmed in this study (Supplementary Materials Figure S4A,B). Further immunofluorescence analyses with anti-rOlyA antibodies demonstrated that after 75 min of exposure of HIB-1B cells to rOlyA, it was internalized, reached the cytoskeleton and colocalized with cellular β-tubulin (Figure 6A,B and Supplementary Materials Figure S6-Video S1).

The interaction between rOlyA and β-tubulin remained stable after 12 h and 14 h of treatment (Figure 6 C,D). Stochastic optical reconstruction microscopy (STORM) imaging showed tight clustering of these two proteins after 14 h of treatment with rOlyA, indicating the molecular interaction between the two (Figure 6F).

Our next objective was to test whether the β-tubulin-binding ability of W28A and W96A mutants is affected. We immunostained both rOlyA and β-tubulin to determine the ability of rOlyA and its mutants to interact with β-tubulin during the time span of 14 h. The rOlyA-treated cells were imaged in a confocal microscope with fine z-stack sampling (0.1 µm step size) to produce 3D images of stained cells (see Figure 6E), demonstrating tight interaction between the two proteins.

We then analyzed the ability of the mutants and OlyA6-mCherry to bind to β-tubulin in cells by measuring colocalization between them. rOlyA mutants lost their ability to efficiently penetrate the cell, and we therefore analyzed their interaction with tubulin ex vivo using β-tubulin in a solid phase ELISA (see Figure 6G). Stable binding between W96A and β-tubulin was slightly lower relative to rOlyA, whereas W28A demonstrated almost negligible binding. rOlyA and β-tubulin solid-phase binding was dose-dependent (data not shown). When W28A was exposed to β-tubulin solid phase, no dose-dependent binding was detected, and the same was observed with OlyA6-mCherry (Figure 6G, bottom panel). Our combined results indicated that W28 is involved not only in lipid raft binding but also in β-tubulin binding. This assumption was further reinforced by the fact that the β-tubulin-binding ability of OlyA6-mCherry, in which W28 points toward the mCherry tag, was completely abolished.

Overall, these results indicate the candidate sites that are important for cytoskeleton interaction and binding and that might be involved in this protein’s activity.

### 3.8. Modeling rOlyA in Complex with β-Tubulin 

In this section, we refer to both aegerolysin proteins—OlyA6 and rOlyA—as rOlyA, because they are 99% identical in their amino acid sequence and have the same secondary and tertiary structures. To build their model, we used the resolved crystal structure of OlyA6 [15]. To predict the binding mode of rOlyA with β-tubulin and to investigate the role of mutated residue W28A in this interaction, protein–protein docking simulations were conducted. αβ-Tubulin dimers shift from the ”bent” conformation observed for free dimers in solution to the “straight” conformation required for incorporation into the microtubule lattice. Crystal structures of β-tubulin in both bent and straight conformations are available in the Protein Data Bank [24]. We previously showed that the addition of rOlyA to paclitaxel-treated cells restores their initial morphology, whereas this does not happen with colchicine-treated cells [2]. Since paclitaxel stabilizes microtubules, whereas colchicine destabilizes them, rOlyA is assumed to interact with β-tubulin in the straight conformation. Therefore, docking was performed between rOlyA and β-tubulin, as extracted from the αβ-tubulin dimer in the straight conformation (PDB ID: 1TUB). We selected the best pose among 30 generated docking poses, considering the ranking order (cluster population) and taking into consideration that, if rOlyA is expected to bind to microtubules, it cannot bind to regions of β-tubulin that are involved in heterodimerization with α-tubulin.

**Figure 7:**
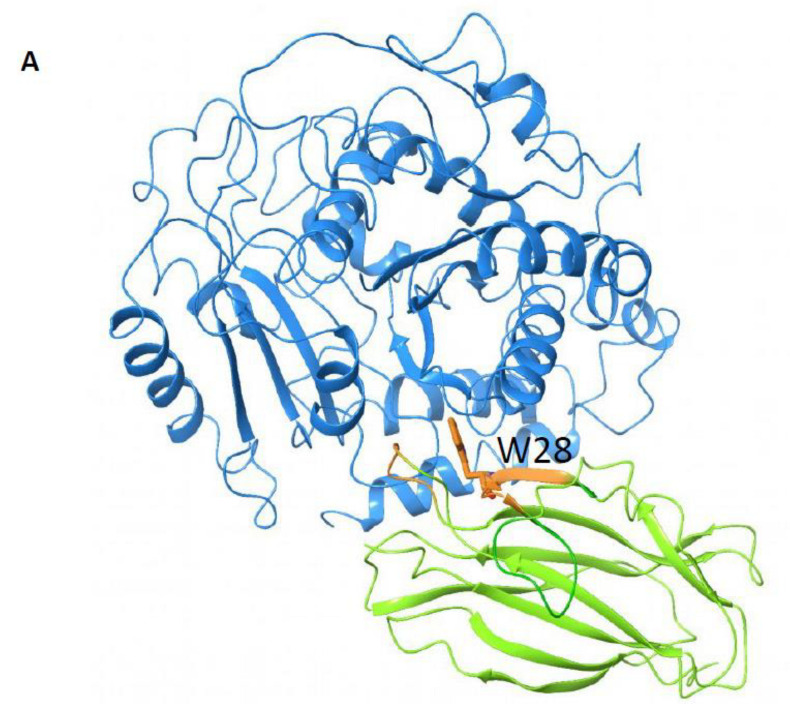

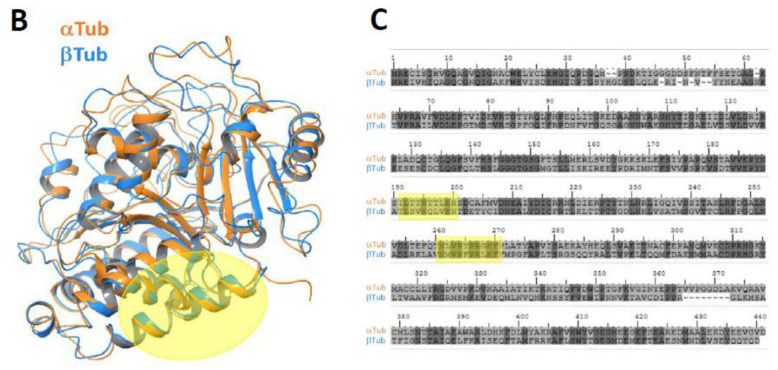
(**A**) Modeling rOlyA with protein–protein docking. Docking pose of rOlyA (in green) with β-tubulin (in blue). The sites overlapping with the region predicted to be “masked” by mCherry are colored in orange, W28 is represented as orange sticks. (**B**) Three-dimensional (3D) superposition of α-tubulin (PDB ID: 1TUB, chain A) and β-tubulin (PDB ID: 1TUB, chain B), as generated with the superposition tool available in Maestro. (**C**) Sequence alignment between α-tubulin (PDB ID: 1TUB, chain A) and β-tubulin (PDB ID: 1TUB, chain B). Regions predicted to be involved in rOlyA interaction are highlighted in yellow.

The selected pose (Figure 7A–C) suggested that W28 interacts with leucine residues L194 and L195 of β-tubulin. The rOlyA surface interacting with β-tubulin partially overlaps with the predicted interface interacting with mCherry in the OlyA6-mCherry complex (residues 26–29 and 67–70, shown in orange in Figure 7B).

### 3.9. α-Tubulin Interaction with rOlyA, Its Mutants and OlyA6-mCherry

α-Tubulin shares 40% amino acid sequence identity with β-tubulin. To detect the proteins that can interact with α-tubulin, sandwich ELISA was performed. rOlyA and its mutants in concentrations of 0.5 ng mL^−1^ were added to immobilized α-tubulin in the solid phase and OlyA6-mCherry at 1.5 ng mL^−1^. We found that rOlyA interacts with α-tubulin as efficiently as with β-tubulin (Figure 6G). W96A and OlyA6-mCherry interacted with α-tubulin at lower efficiency, in contrast to the results from the β-tubulin solid-phase interaction (Figure 6G). The W28A mutant lost its ability to bind to both α- and β-tubulin (Figure 6G), even at higher concentrations (1 ng mL^−1^—data not shown). Interestingly, the β-tubulin site predicted to be responsible for rOlyA binding coincided with a highly conserved region between the two proteins (Figure 7B).

### 3.10. Visualization of Colocalization of Tubulin Filaments and the Protein of Interest in HIB-1B Cells

**Figure 8:**
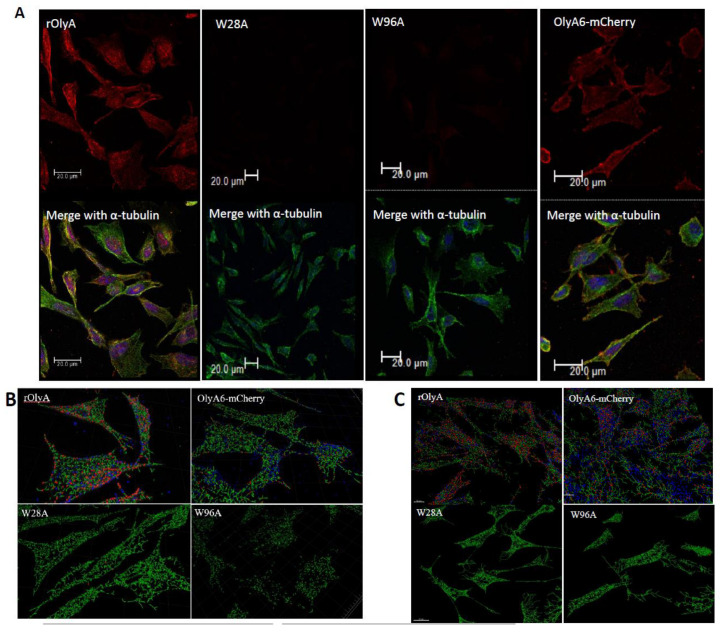

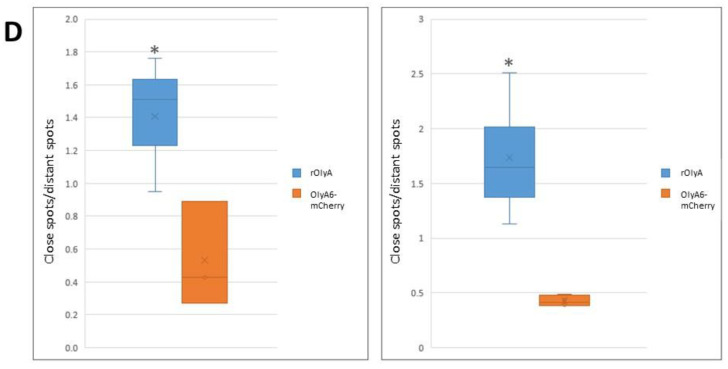
Colocalization of rOlyA and α-tubulin in HIB-1B cells at 150 min post-treatment. HIB-1B cells were treated with rOly, W28A, W96A 10 μg mL^−1^ and OlyA6-mCherry 30 μg mL^−1^. Confocal microscopy imaging (**A**) and spatial distribution of the visualization by Imaris analysis (**B**–**D**). (**A**) Represented distribution in treated preadipocytes and α-tubulin interaction from confocal microscopy. Fixed cells were stained with anti-rOlyA antibodies (red, top panel), anti-α-tubulin antibodies (green) and DAPI (bottom panel—merged images). Intracellular microtubules (MTs) were determined by z-stack acquisition in a confocal microscope. Scale bars = 20 μm. (**B**) Imaris analysis represents protein interaction with α-tubulin filaments in treated cells. rOlyA and OlyA6-mCherry colocalize with α-tubulin filaments, whereas mutants W28A and W96A do not. (**C**) Imaris analysis of protein interaction with β-tubulin filament in treated cells. Representative cells were selected from a random field of view on the coverslip. The resulting images were analyzed with Imaris software. Finely spaced confocal stacks were acquired to reconstruct a 3D cell volume. Deconvolved stacks were analyzed via Imaris software to generate spots corresponding to the individual rOlyA or OlyA6-mCherry spots and to analyze the colocalization with α/β-tubulin filaments. (**D**) Efficiency of interaction with α-tubulin (left panel) and β-tubulin (right panel), graphic representation. Analysis performed with Imaris software to generate spots corresponding to α-tubulin- and rOly-containing protein from confocal images. Quantification of the cytoplasmic foci that contain both α-tubulin and rOlyA (close—colocalized, distant—not colocalized). The proportions of the total spots in each cell that were colocalized, and those that were not colocalized, are presented in a box-and-whisker plot. * *p*-value < 0.0001.

We visualized α-tubulin filaments in HIB-1B cells treated with rOlyA, rOlyA mutants or OlyA6-mCherry. We used Imaris software to quantify the ratio of close spots to distant spots showing the proteins of interest. The ratio of these spots’ calculations was based on the relative distance between rOlyA or OlyA6-mCherry and α/β-tubulin filaments in HIB-1B cells. We calculated the relative distance between rOlyA or OlyA6-mCherry spots and α- or β-tubulin filaments in HIB-1B cells at 90 min post-treatment. Representative cells were selected from a random field of view on the coverslip, and 3D confocal stacks of fluorescence were background subtracted and deconvoluted. The resulting images were analyzed with Imaris software (version 8.4.1; Bitplane AG, Oxford Instruments, UK). rOlyA and OlyA6-mCherry colocalized with α-tubulin filaments in the cell. Mutants W28A and W96A did not colocalize with the α-tubulin filaments (Figure 8). The total number of spots was generated by Imaris for each cell type by treatment (Table 1).

The ratio between close protein spots and distant protein spots was calculated to obtain the rate of rOlyA and tubulin colocalization. The relative distance of rOlyA and α/β-tubulin filaments in HIB-1B cells was calculated by Imaris software to quantify close/distant proteins. The W28A mutant did not colocalize with α-tubulin filaments, whereas mutant W96A minimally colocalized with α-tubulin filaments.

In general, we found similar rOlyA and OlyA6-mCherry configurations of cytoplasmic foci in rOlyA- and OlyA6-mCherry-treated cells (Figure 8B). We analyzed the proportion of cytoplasmic foci containing spots in the treated cells. Results showed that rOlyA has a higher number of colocalized spots with α- and β-tubulin than OlyA6-mCherry (Table 1, Figure 8 B,C). In addition, analysis of cells treated with the mutants revealed very low numbers of spots per cell. Specifically, in W96A-treated cells, this was due to the abolishment of its cell internalization. In the W28A-treated cells, the number of intracellular spots was higher than in the W96A-treated cells but still very low compared to rOlyA- and OlyA6-mCherry-treated cells.

## 4. Discussion

Our previous research demonstrated that the oyster mushroom-derived recombinant aegerolysin protein rOlyA binds to adipocyte lipid rafts, is internalized into the cell via caveolin-1-dependent endocytosis and induces brown adipocyte differentiation through activation of PGC-1α and stimulation of mitochondrial biogenesis [2]. The goal of the present study was to identify the rOlyA residues involved in these activities. Moreover, our interest was to assess the main cellular components involved in rOlyA cell internalization and its associated biological activity.

Microtubules play a major role in the intracellular trafficking of vesicles in cells. We previously demonstrated the interaction of rOlyA with β-tubulin and its association with preadipocytes’ differentiation process [2]. Here, we explored the role of microtubules in rOlyA cell internalization and cellular activity.

Our results showed that rOlyA and β-tubulin colocalization require conservation of specific residues and no allosteric disturbance of rOlyA. rOlyA progresses toward the nucleus and, after 24 h, is mostly localized proximally to it. Detailed observation of rOlyA and β-tubulin intracellular colocalization at different time points indicated the putative involvement of β-tubulin in protein shuttling from the membrane to the nucleus.

To further explore OlyA’s interaction with β-tubulin, we designed and analyzed the function of rOlyA mutants and the OlyA6-mCherry construct. Several tryptophan residues are involved in membrane–protein interactions and SM/cholesterol binding in the highly homologous aegerolysin protein PlyA2 [9]. Therefore, we designed rOlyA mutants by introducing tryptophan-to-alanine point mutations in rOlyA at positions 28, 92, 96 and 112. These residues are highly conserved in the aegerolysin proteins produced by *Pleurotus* mushrooms (PlyA2, PlyA, EryA, OlyA, OlyA6). The mutant W96A completely lost its ability to be internalized into the cell, whereas internalization of the mutant W28A was reduced (Supplementary Materials Figure S3). Furthermore, W96A retained its ability to bind β-tubulin, although it was slightly lower compared to rOlyA. The position at which W96 was involved in the membrane interaction and cell penetration is reported in Supplementary Materials Figure S3. Importantly, sandwich ELISAs showed that β-tubulin binding was abolished in the W28A mutant (Figure 6G).

The fluorescent recombinant protein OlyA6-mCherry is a potential marker for membrane microdomains enriched in SM and cholesterol that fulfill the structural requirements for lipid rafts [5]. Impaired ability of OlyA6-mCherry to interact with microtubular filaments did not completely disrupt its full biological function, but it did markedly reduce it. We evaluated the biological activity of OlyA6-mCherry in HIB-1B adipocytes and tested OlyA6-mCherry binding to β-tubulin by ELISA. OlyA6-mCherry was capable of inducing some of the differentiation markers but could not bind to β-tubulin. Differences between rOlyA and rOlyA6-mCherry binding to β-tubulin were also observed by co-immunostaining and analysis with Imaris software. We found no binding of OlyA6-mCherry to β-tubulin in the solid phase, but we detected colocalization with the aid of confocal imaging. However, OlyA6-mCherry showed specific binding to α-tubulin in a solid-phase ELISA. In cells, α/β-tubulins form integrated filaments [25], and this could explain the observed colocalization of OlyA6-mCherry with microtubules in vitro. Our results suggest that progressive cellular activity may be ”masked” by the mCherry tag in OlyA6-mCherry, as evidenced by the reduced expression of the genes involved in the differentiation process and progressive cell proliferation. All of these lost activities are associated with reduced interaction with β-tubulin. We concluded that the OlyA6 region covered by mCherry is the region involved in β-tubulin-binding specificity. To complement the experimental results and determine the region of OlyA6 covered by mCherry, we further performed a structural prediction of the OlyA6-mCherry complex.

PGC-1α plays key roles in the stimulation of oxidative capacity, regulation of mitochondrial biogenesis and expression of mitochondrial oxidative enzymes [23]. PGC-1α exhibits its highest expression in brown adipose tissue, where it exerts some of its most well-established roles [26]. Initially discovered for its ability to induce mitochondrial biogenesis and adaptive thermogenesis in brown adipocytes, PGC-1α has been shown to coordinate the expression of thermogenic genes [26]. Humans with diabetes have reduced PGC-1α levels in their adipose tissue, which may contribute to the pathogenesis of the disease [27,28,29]. For these reasons, promising therapeutic strategies have been developed based on novel pathways associated with PGC-1α activation and its target genes. Here, we found that rOlyA upregulates *PGC-1α* expression, whereas the W96A and W28A mutants did not. *PGC-1α* gene expression is highly inducible by rOlyA and, accordingly, we demonstrated enhanced mitochondrial biogenesis and activity in these treated cells. We analyzed genes coding for mitochondrial activity: *Cox5b* (a component of cytochrome c oxidase, the last enzyme in the mitochondrial electron transport chain that drives oxidative phosphorylation), *ATPase_B2* (the mitochondrial ATPase_B2 that plays a pivotal role in maintaining cellular ATP levels in eukaryotic organisms) and *ND5* (mitochondrial gene *ND5* is an essential subunit of the mouse respiratory NADH dehydrogenase (complex I)). In both rOlyA- and Oly6-mCherry-treated HIB-1B preadipocytes, the levels of *PGC-1α* gene expression were elevated 24 h post-treatment; however, they only remained significantly elevated in rOlyA-treated cells for 48 h post-treatment.

The present results, added to our previous studies [2,8,30], further contribute to our understanding of rOlyA activity and the role of cytoskeletal proteins in this important process. Here, we clearly demonstrate that cellular microtubules play a key role in rOlyA-mediated effects in HIB-1B preadipocyte cells; i.e., induction of lipid droplets formation and increased cellular respiration. rOlyA-mediated effects directly affect metabolic diseases [2,8,30] and cancer-associated processes [8]. In addition, we present novel data on the role of certain residues of rOlyA that are essential for the interaction of this protein with α/β-tubulin filaments.

## Figures and Tables

**Table 1:** Imaris analysis: protein interaction with α/β-tubulin filaments after 150 min of treatment. The ratio between close protein spots and distant protein spots was calculated to obtain the rate of OlyA-containing proteins and tubulin colocalization. Mutant W28A did not colocalize with α-tubulin filaments; W96A, with abolished cell-internalization ability, showed minimal colocalization with α-tubulin filaments.

		Average	Average	Average	Average
		rOlyA	OlyA6-mCherry	W28A	W96A
α-tubulin	Spots/cell	297.4	309.82	2.82	2.98
	Close spots	3412.67	3039	110.5	16.33
	Distant spots	2342.67	5823	47	34.33
	Close spots/distant spots	1.41	0.53		
		Average	Average	Average	Average
β-tubulin	Spots/cell	191.08	248.41	0	3.5
	Close spots	4887	1892.5	0	22.5
	Distant spots	3108.25	4495.25	0	49
	Close spots/distant spots	1.74	0.43		

## Supplementary Materials

The following supporting information can be downloaded at: https://www.mdpi.com/article/10.3390/biomedicines10102442/s1, Figure S1: Western blot with anti-rOlyA antibodies for 300 ng purified mutant proteins W28A and W96A after Superdex 75 re-chromatography compared to rOlyA protein, confirming the presence of the protein of interest in the eluted fractions. Elution tubes W96A-3, W96A-4, W96A-5, W96A-6 and W28A-7 contain mutant protein, Figure S2: Dose-response effect of control and mutant proteins on lipid droplet accumulation in HIB-1B cells, Figure S3: Presence of intracellular protein rOlyA and mutant proteins in treated HIB-1B cells, Figure S4. (A) Super-resolution STORM imaging of rOlyA and caveolin-1 protein interaction 14 h post-rOlyA treatment of preadipocytes. (B) Colocalization of rOly and caveolin-1 in HIB-1B preadipocytes 2 h after treatment, Figure S5: Intracellular distribution of rOlyA in fixed rOlyA-treated HIB-1B control preadipocytes or MβCD-treated cells (HIB-1B cells were previously treated with the endocytic pathway inhibitor MβCD at 1.25 mM for 4.5 h). Figure S6-Video 1. Colocalization of rOly and α-tubulin in HIB-1B cells 150 min post-treatment. Represented distribution of rOly (red) in treated preadipocytes and α-tubulin (green) interaction. Leica 3D tool. Fixed cells were stained with anti-α-tubulin and anti-rOlyA antibodies. Intracellular MT were determined by z-stack acquisition in a confocal microscope. Scale bar = 5 μm. Representative cells chosen from the entire coverslip are displayed for clarity. HIB-1B cells were treated with 10 μg ml-1 rOlyA.

## Abbreviations

Ostreolysin A6 (OlyA6), recombinant OlyA (rOlyA), pleurotolysin A2 (PlyA2), sphingomyelin (SM), isopropyl-1-thio-β-D-galactopyranoside (IPTG), size-exclusion chromatography (SEC), circular dichroism (CD), stochastic optical reconstruction microscopy (STORM).
