# Supplementary material for: Critical Sites on Ostreolysin Are Responsible for Interaction with Cytoskeletal Proteins
Source: Biomedicines. 2022 Sep 30;10(10):2442. doi: 10.3390/biomedicines10102442 (PMC9598724; doi:10.3390/biomedicines10102442)
Supplement: Supplementary file 1 [file biomedicines-10-02442-s001.zip › biomedicines-1918766-supplementary.pdf]

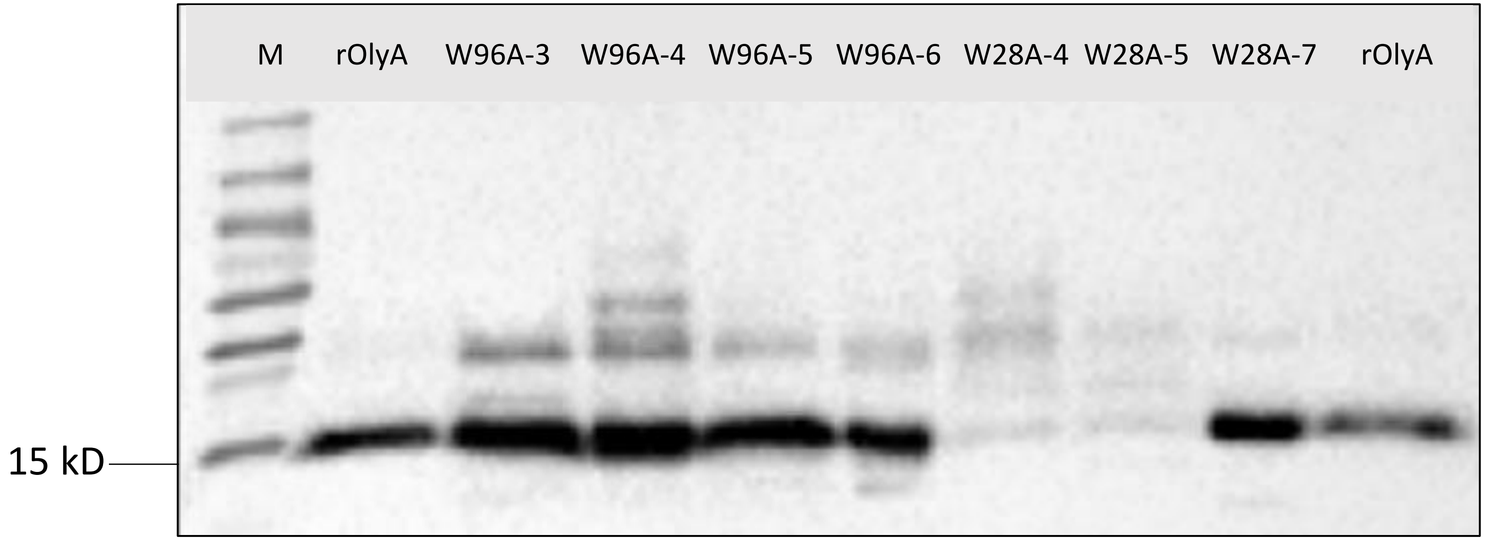

Supplementary Fig. S1

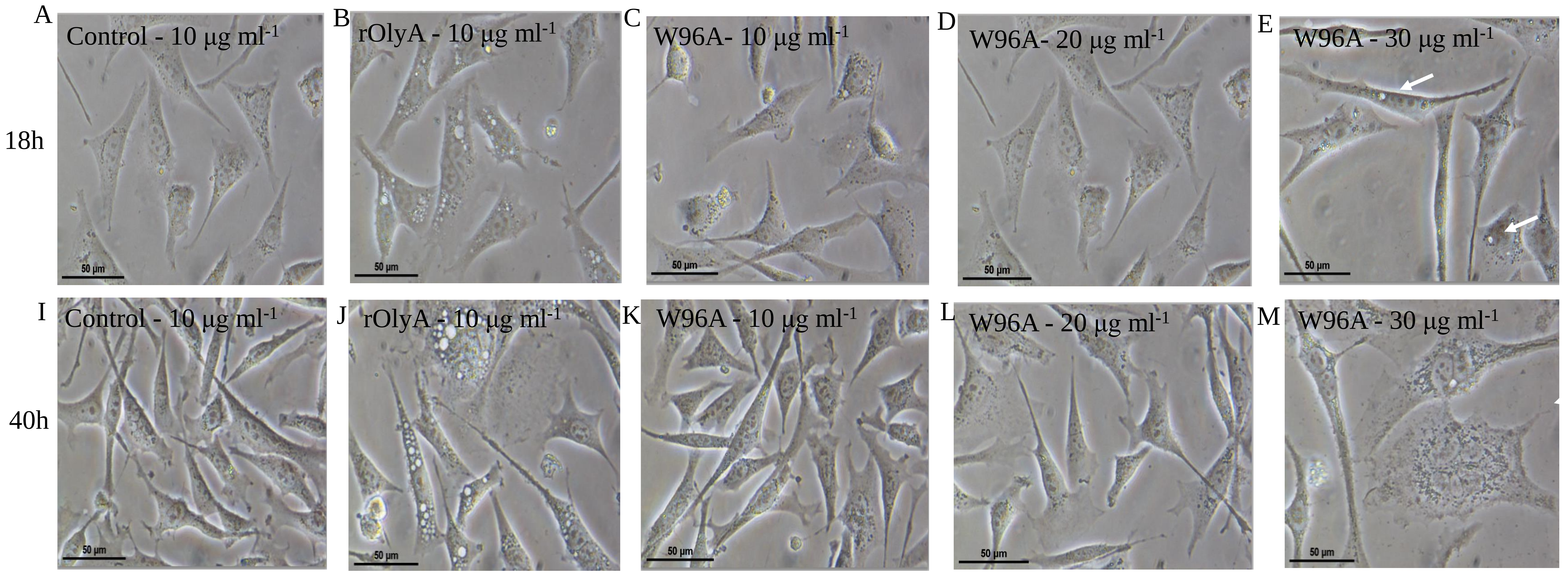
A
Control - 10 µg ml-1
B
rOlyA - 10 µg ml-1
C
W96A- 10 µg ml-1
D
W96A- 20 µg ml-1
E
W96A - 30 µg ml-1
18h
I
Control - 10 µg ml-1
J
rOlyA - 10 µg ml-1
K
W96A - 10 µg ml-1
L
W96A - 20 µg ml-1
M
W96A - 30 µg ml-1
40h
50 µm

Supp. Fig. S2

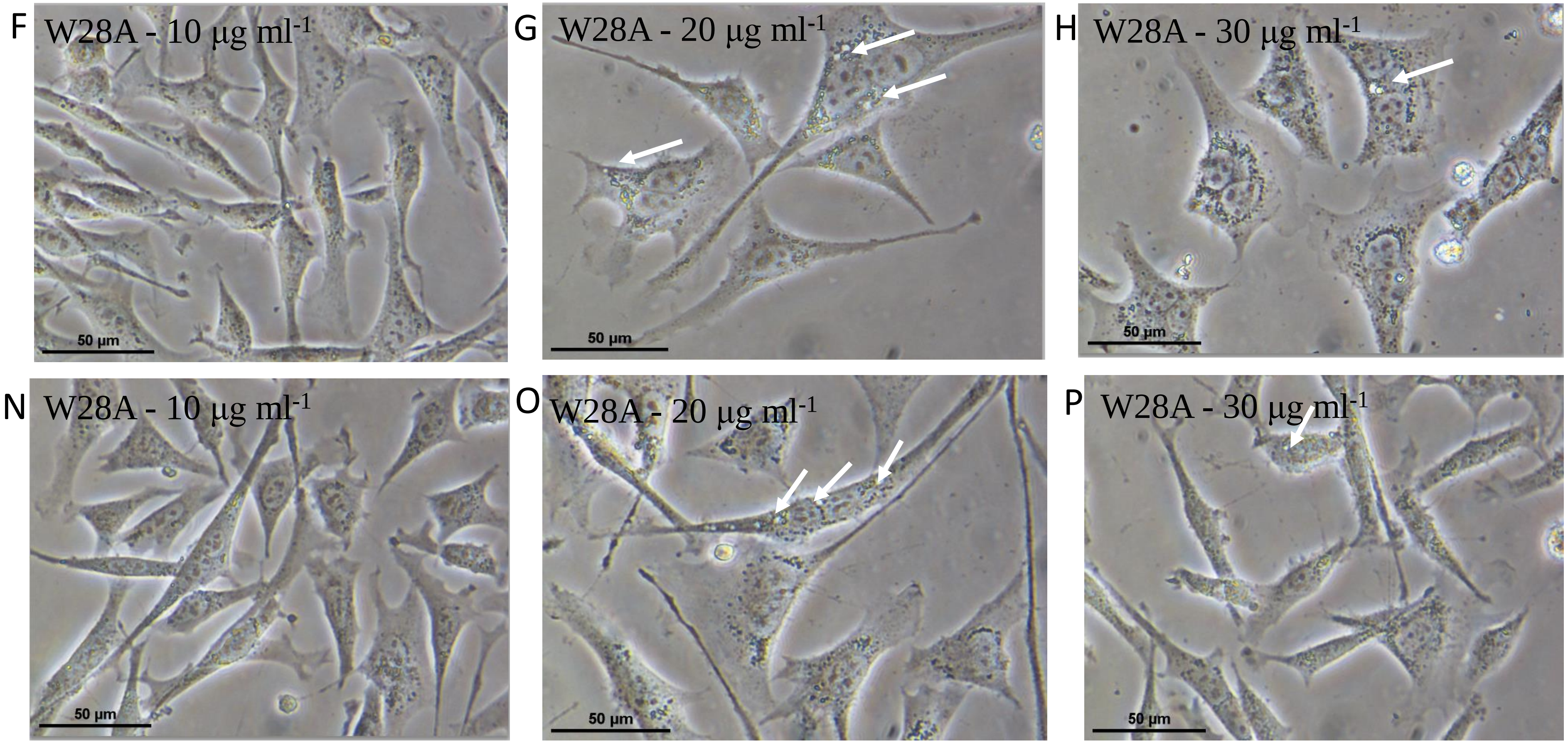
F
W28A - 10 µg ml-1
50 µm
G
W28A - 20 µg ml-1
50 µm
H
W28A - 30 µg ml-1
50 µm
N
W28A - 10 µg ml-1
50 µm
O
W28A - 20 µg ml-1
50 µm
P
W28A - 30 µg ml-1
50 µm

Supp. Fig. S2

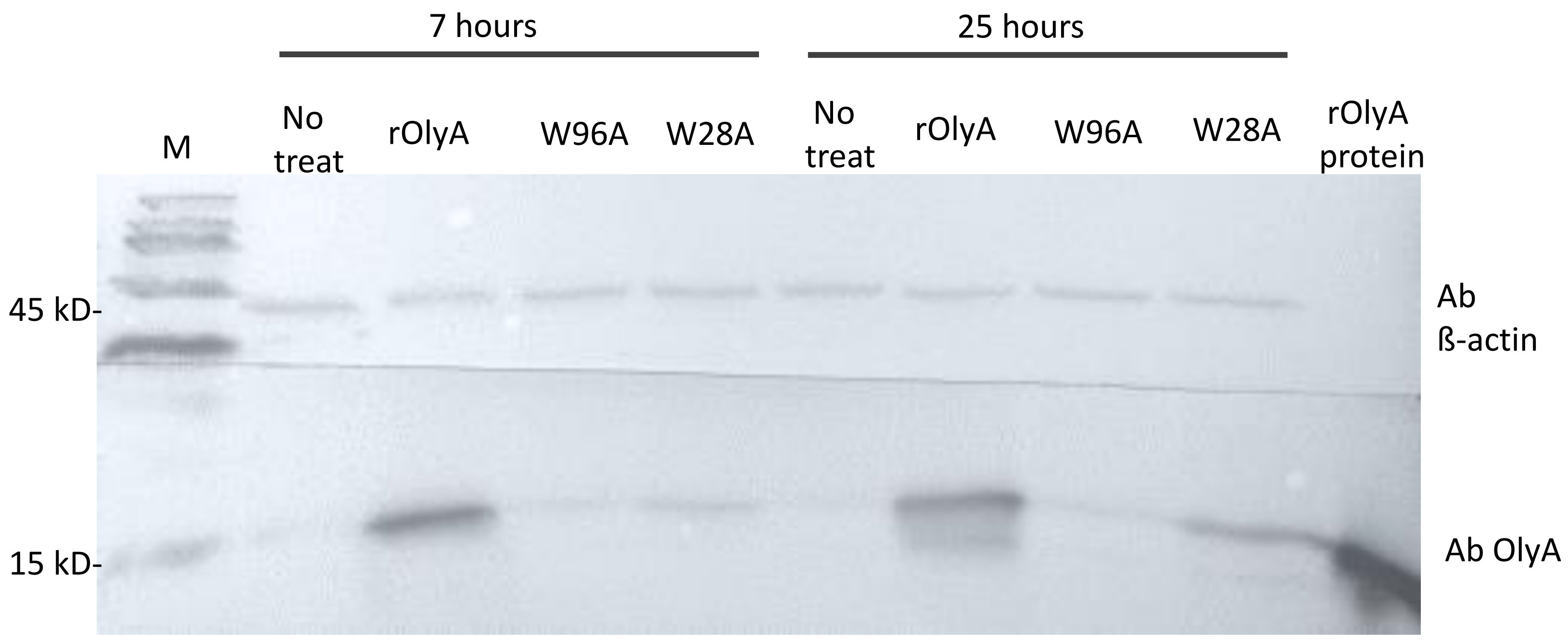
7 hours
25 hours
M
No treat
rOlyA
W96A
W28A
No treat
rOlyA
W96A
W28A
rOlyA protein
45 kD-
15 kD-
Ab ß-actin
Ab OlyA

Supp. Fig. S3

A

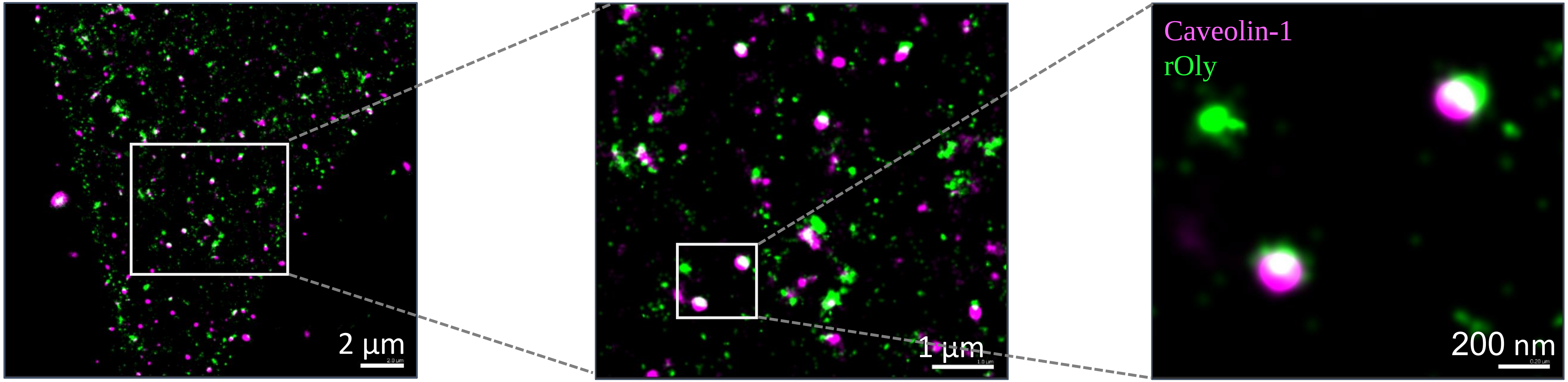

Supp. Fig. S4

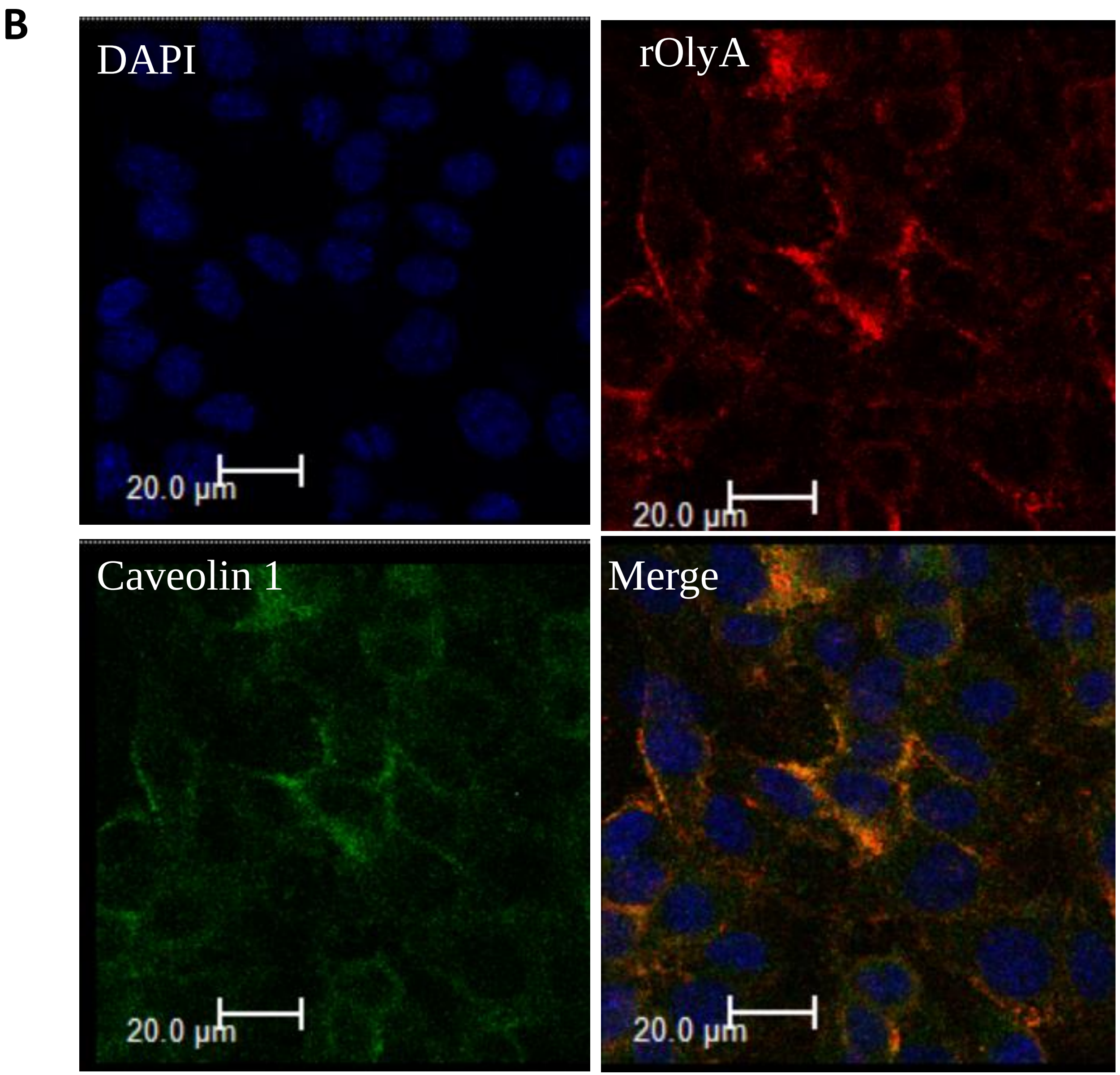

Supp. Fig. S4B

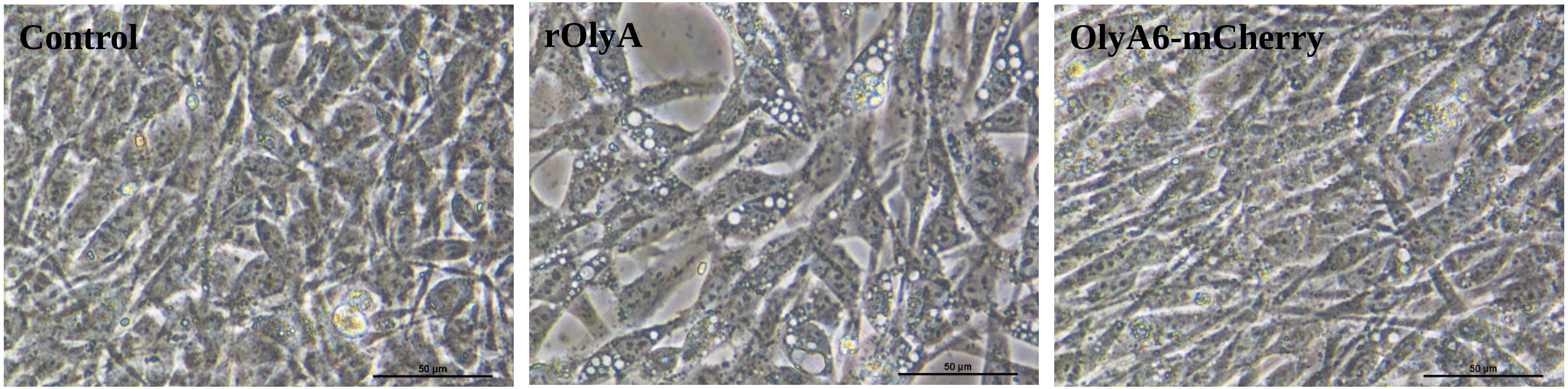

Supp. Fig. S5

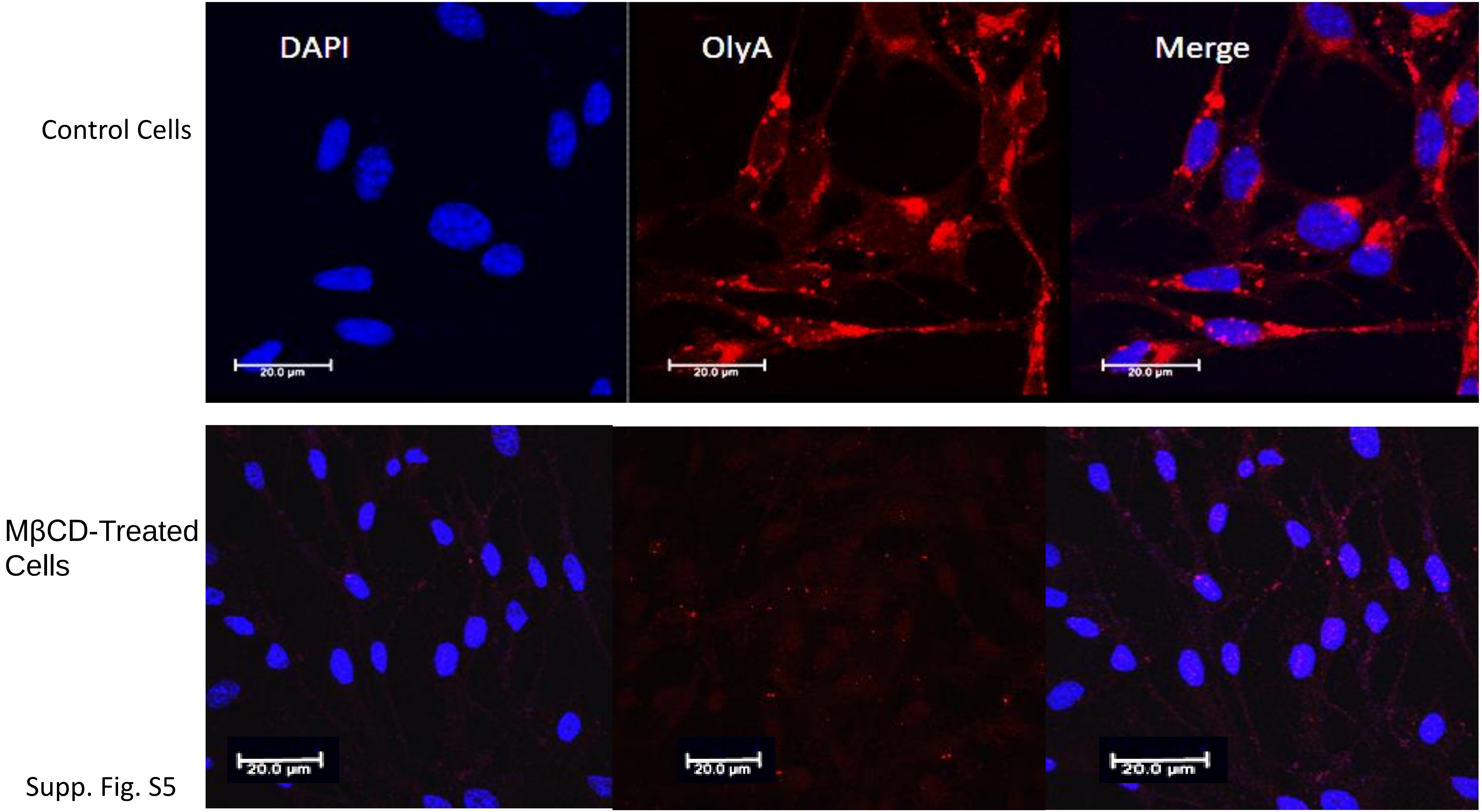
DAPI
OlyA
Merge
Control Cells
MβCD-Treated Cells
20.0 μm
20.0 μm
20.0 μm
20.0 μm
20.0 μm
20.0 μm

Supp. Fig. S5

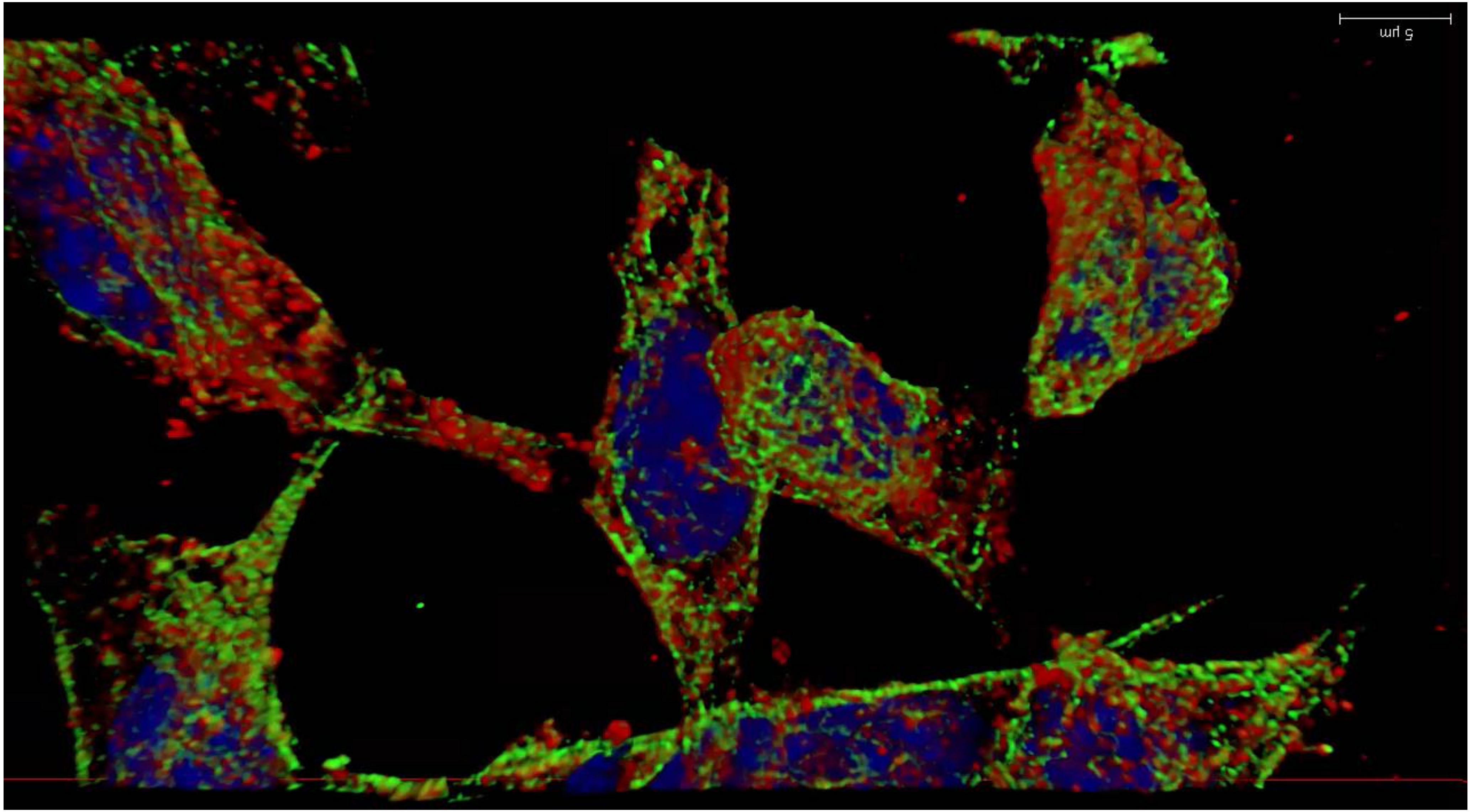

Supp Fig.
S6 Video
S1
